# De novo design of a nanoregulator for the dynamic restoration of ovarian tissue in cryopreservation and transplantation

**DOI:** 10.1186/s12951-024-02602-5

**Published:** 2024-06-11

**Authors:** Min Jiang, Guo-Hui Zhang, Yuan Yu, Yu-Hong Zhao, Jun Liu, Qin Zeng, Meng-Yue Feng, Fei Ye, Dong-Sheng Xiong, Li Wang, Ya-Nan Zhang, Ling Yu, Jia-Jing Wei, Li-Bing He, Weiwei Zhi, Xin-Rong Du, Ning-Jing Li, Chang-li Han, He-Qiu Yan, Zhuo-Ting Zhou, Yang-Bao Miao, Wen Wang, Wei-Xin Liu

**Affiliations:** 1 School of Medicine and Life Sciences, Chengdu University of Traditional Chinese Medicine, Chengdu, 611137 Sichuan China; 2 Key Laboratory of Reproductive Medicine, Sichuan Provincial Maternity and Child Health Care Hospital, The Affiliated Women’s and Children’s Hospital of Chengdu Medical College, Chengdu, 610045 China; 3 School of Clinical Laboratory Medicine, Chengdu Medical College, Chengdu, 610083 China; 4 Department of Haematology, Sichuan Academy of Medical Sciences & Sichuan Provincial People’s Hospital, School of Medicine, University of Electronic Science and Technology of China, Chengdu, 610000 China

**Keywords:** Ovarian tissue cryopreservation and transplantation, Nanoregulators, Nanoparticle, Ovarian system, Reproductive system, Transplantation

## Abstract

The cryopreservation and transplantation of ovarian tissue underscore its paramount importance in safeguarding reproductive capacity and ameliorating reproductive disorders. However, challenges persist in ovarian tissue cryopreservation and transplantation (OTC-T), including the risk of tissue damage and dysfunction. Consequently, there has been a compelling exploration into the realm of nanoregulators to refine and enhance these procedures. This review embarks on a meticulous examination of the intricate anatomical structure of the ovary and its microenvironment, thereby establishing a robust groundwork for the development of nanomodulators. It systematically categorizes nanoregulators and delves deeply into their functions and mechanisms, meticulously tailored for optimizing ovarian tissue cryopreservation and transplantation. Furthermore, the review imparts valuable insights into the practical applications and obstacles encountered in clinical settings associated with OTC-T. Moreover, the review advocates for the utilization of microbially derived nanomodulators as a potent therapeutic intervention in ovarian tissue cryopreservation. The progression of these approaches holds the promise of seamlessly integrating nanoregulators into OTC-T practices, thereby heralding a new era of expansive applications and auspicious prospects in this pivotal domain.

**Graphical Abstract:**

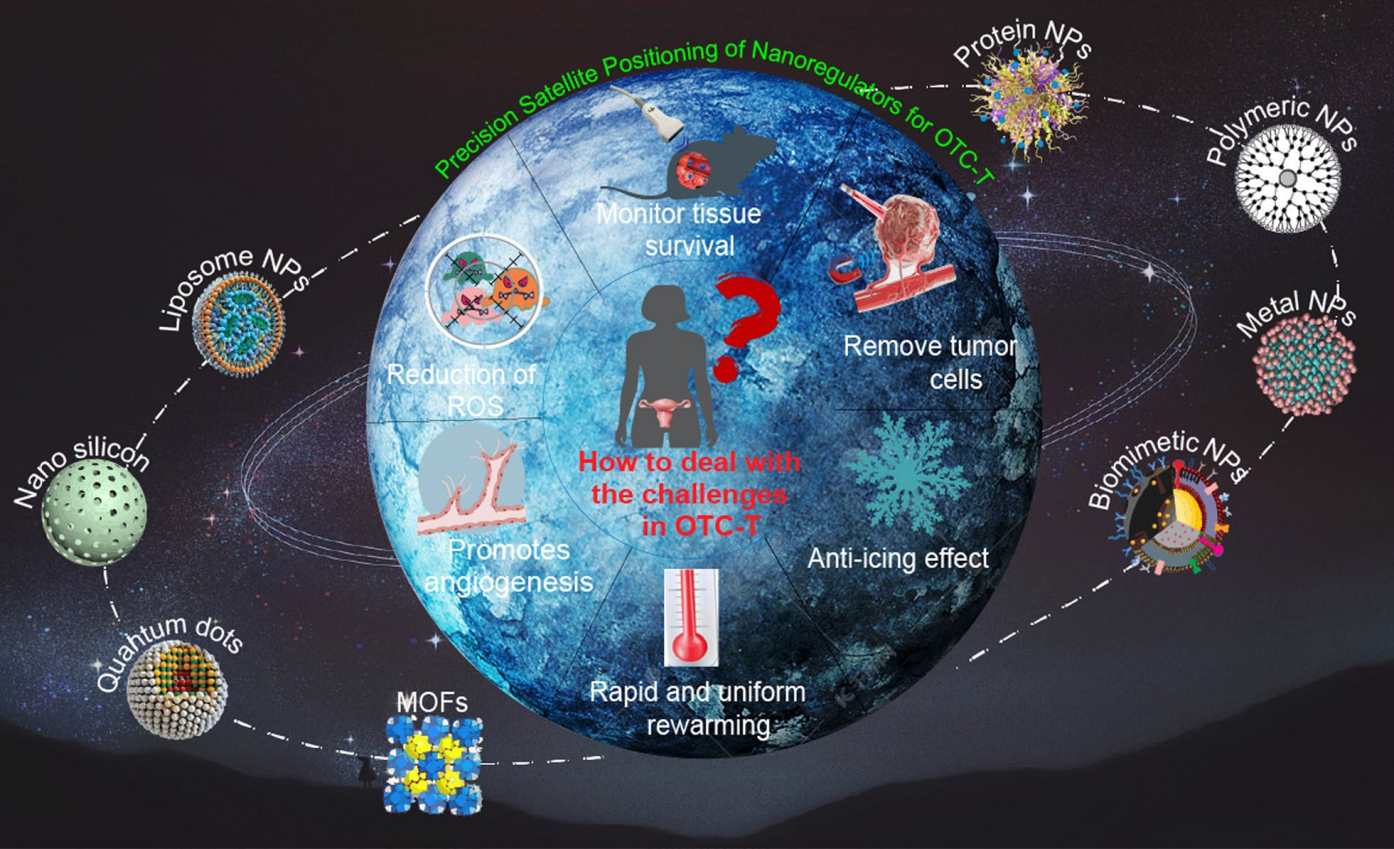

## Introduction

In the intricate tapestry of female physiology, the ovaries assume a central role. Their production of indispensable hormones regulates the female reproductive endocrine system and contributes to overall health maintenance. Regrettably, the ovaries are susceptible to irreversible damage from various factors, with radiation and chemotherapy exerting particularly severe impacts [1–4]. Recent advancements in ovarian tissue cryopreservation and transplantation techniques have emerged as a beacon of hope, attracting considerable attention, especially among cancer patients. These techniques not only preserve fertility, fulfilling reproductive desires, but also uphold endocrine functions, offering novel avenues for preventing premature ovarian failure [5–7].

For prepubescent females facing cancer, those urgently requiring radiotherapy or chemotherapy, or postpubescent females intolerant to ovarian stimulation, this technology stands as their sole recourse to retaining fertility, witnessing widespread global application [8–10]. However, the evolution of this technology encounters significant hurdles, including the toxicity of cryoprotectants [11–13], cellular and tissue damage from ice crystal formation [14], angiogenesis challenges [15, 16], oxidative stress from reactive oxygen species [17, 18], and the risk of tumor cell contamination [19, 20]. Consequently, innovative solutions to these challenges are imperative.

In recent years, nanotechnology has witnessed extensive research and application in biomedicine [21, 22]. Nanoregulators, with their expansive specific surface area and distinctive physical, chemical, mechanical, and biological properties, have become the focus of researchers. With continuous technological strides, nanoregulators have achieved breakthroughs in diverse research fields, notably in early tumor diagnosis and treatment [23], drug encapsulation and delivery [24], and tissue engineering [25]. Their application in female reproduction and health is also expanding [26–28]. However, the integration of nanotechnology into OTC-T encounters formidable challenges. Key concerns include the precise targeting of lesion sites, optimization of ovarian transplantation effectiveness, and the potential for toxicity across various systems, encompassing reproductive and biocompatibility issues [29–31].

To address these challenges, strategies have been developed, encompassing the synthesis and application of nanoregulators [32], optimization of synthesis techniques and materials, and surface modification [33–35]. These strategies aim to mitigate the toxicity of nanomodulators while enhancing their efficacy.

**Fig. 1:**
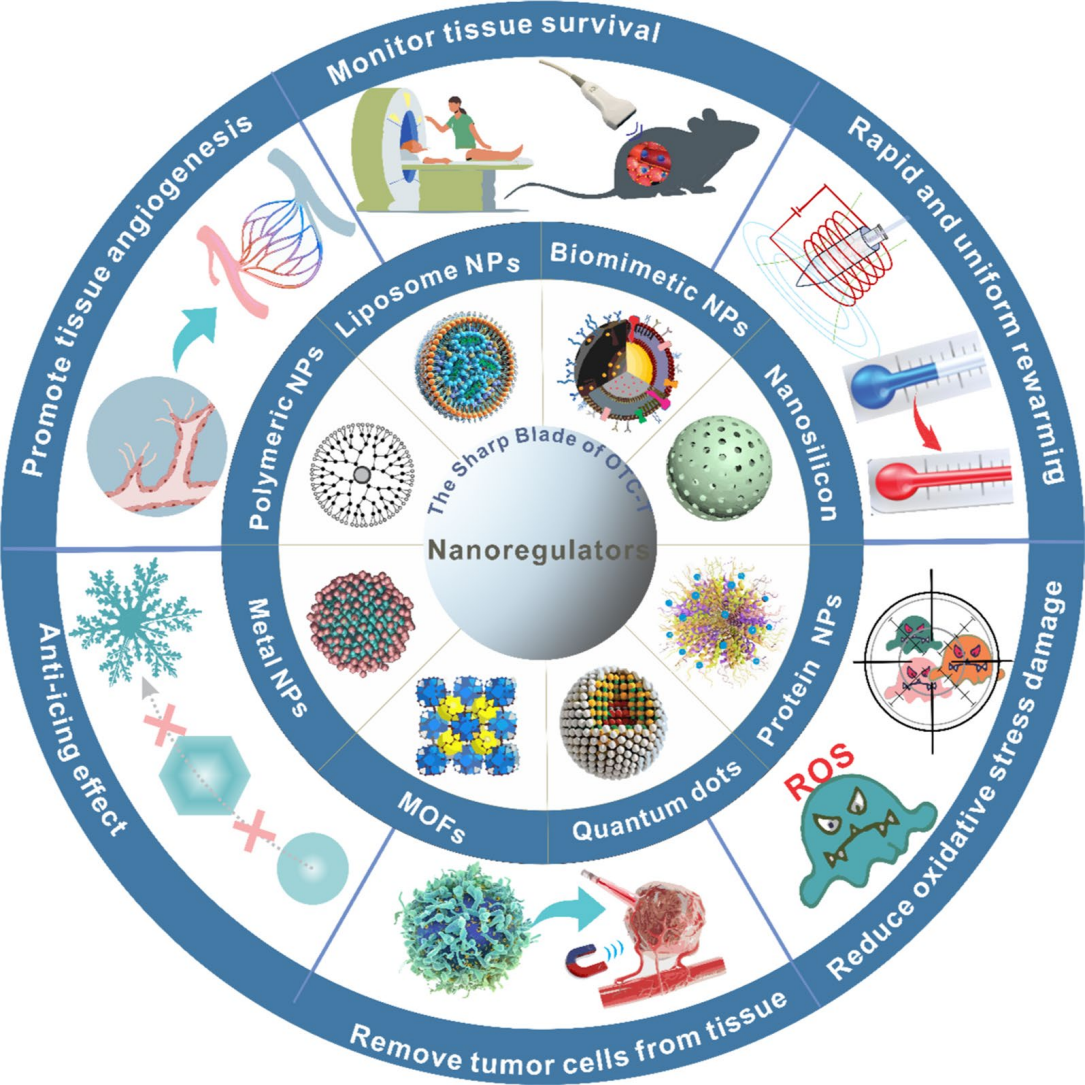
Dynamic repair of OTC-T by nanoregulators. In the process of ovarian tissue cryopreservation and transplantation, nanoregulators effectively inhibit the formation of ice crystals, promote angiogenesis in the transplanted tissues, reduce oxidative stress damage in the tissues, monitor the survival status of the transplanted tissue, and eliminate tumor cells within the transplanted tissue

This review delves into a comprehensive analysis of nanoregulator application in OTC-T (Fig. 1). Leveraging the exemplary performance of nanoregulators in OTC-T and drawing insights from their success in other transplantation fields, we explore their potential applications in overcoming challenges associated with OTC-T. By providing an in-depth review of the ovarian physiological structure and its intricate microenvironment, we establish a foundational framework for understanding the role of nanoregulators in OTC-T. Our exploration of ovarian function aims to deepen the reader’s appreciation of the significance of OTC-T, particularly focusing on how nanotechnology can address challenges in the process.

The review emphasizes the synthesis and application of microbially derived nanoregulator, striving to facilitate a safer and more effective integration of nanoregulators with OTC-T. Additionally, we explore methods to enhance the safety of nanomaterial applications, including surface modification techniques. Furthermore, nanoregulators may offer new material support for constructing artificial ovaries and developing ex vivo culture substrates. We maintain a positive outlook on the application of nanotechnology in these realms, anticipating that, akin to other fields, nanotechnology will bring new hope and possibilities to OTC-T.

## Ovarian tissue cryopreservation and transplantation (OTC-T)

Endowed with distinctive features including a porous and ordered structure, as well as the magnetothermal characteristics of metallic nanoregulators, nanoregulators exhibit significant potential in addressing the challenges associated with OTC-T. These materials hold the promise of reducing ice crystal formation and improving the rewarming process post-cryopreservation, leading to enhanced outcomes in cell revival. A comprehensive understanding of ovarian structure and function, along with a nuanced appreciation of the challenges encountered during the OTC-T process, is imperative for the effective utilization of nanoregulators. This knowledge is pivotal for advancing our understanding of the OTC-T process and the intricate mechanisms through which these materials operate.

### The structure and function of the ovary

The ovary is a complex organ integral to the female reproductive system, playing a pivotal role in various physiological functions [36]. Its intricate structure and multifaceted functions are essential for regulating the female reproductive endocrine system and maintaining overall health. The ovaries produce crucial hormones that govern reproductive processes. However, they are susceptible to irreversible damage from various factors, particularly the harsh impacts of radiation and chemotherapy [37]. Understanding the detailed structure and function of the ovary is fundamental to appreciating its significance and addressing challenges, especially in the context of advancements in reproductive technologies like ovarian tissue cryopreservation and transplantation.

#### Detailed examination of ovarian physiology

**Fig. 2:**
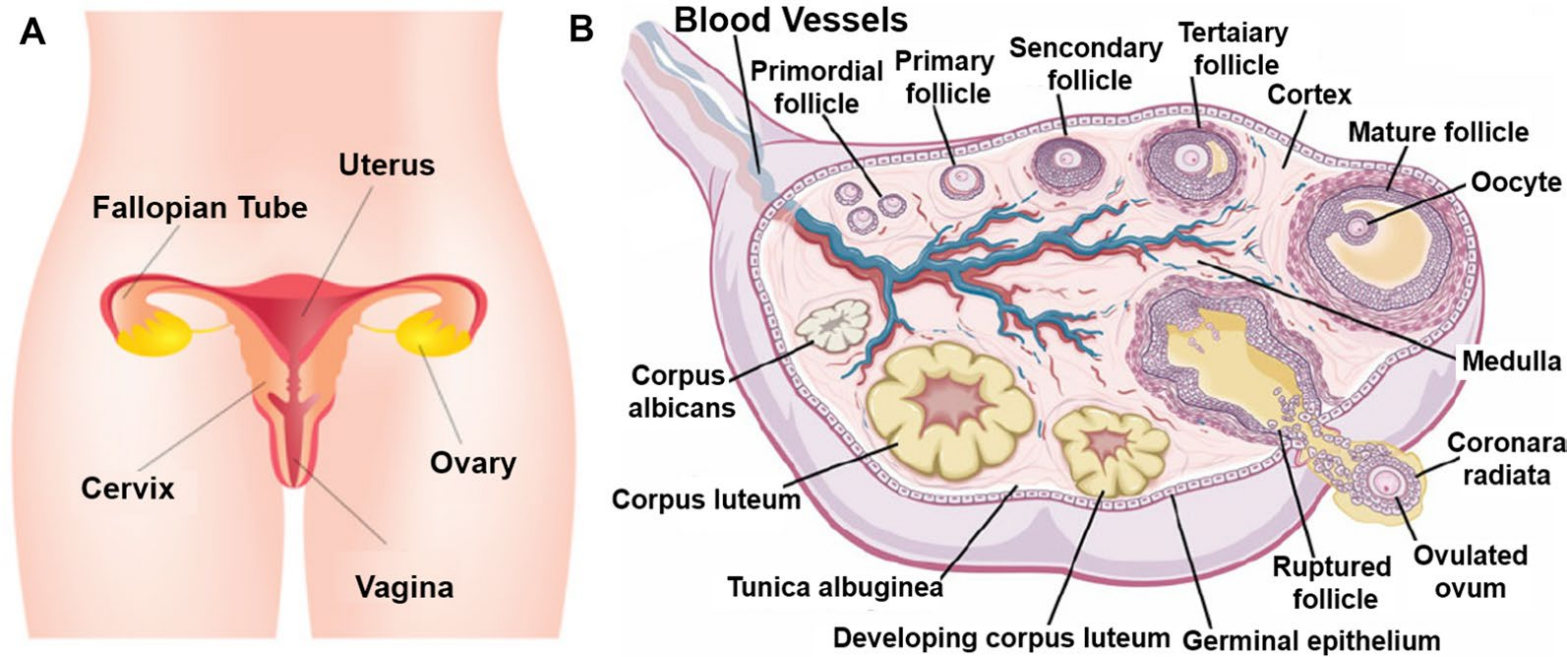
**A** The basic structure of the female reproductive system. **B** Apart from vascular and neural structures within the ovarian stroma, the primary constituents of the ovary include primordial follicles located in the cortex, as well as follicles at various stages of development. These follicles undergo maturation and eventually lead to the formation of ova through the process of ovulation [42]

The ovaries are a pair of solid organs located in the female pelvis, typically oval and flattened in shape [38]. The surface of the ovaries is covered by a layer of simple flat or cuboidal epithelium, a common site for the development of ovarian cancer. Beneath this surface epithelium lies the tunica albuginea, composed of a thin layer of dense connective tissue. The substance of the ovary is divided into an outer cortex and an inner medulla [39]. The cortex is the most functionally significant region of the ovary, as it contains follicles at various stages of development and is the primary site for ovarian tissue cryopreservation and transplantation. The ovarian medulla [40] is made up of loose connective tissue and contains a rich supply of blood vessels, lymphatics, and nerves, providing the cortex with nutrients and facilitating substance exchange (Fig. 2) [41].

The ovaries, as the primary female gonads, play a crucial role in facilitating the development, maturation, and ovulation of follicles. They are also involved in the development and maintenance of female secondary sexual characteristics and overall health [43]. The ovaries secrete over 20 different hormones and growth factors, including estrogens, progesterone, and androgens, which are vital for female reproduction and health [44]. Estrogens have a broad and significant physiological role, promoting the growth and development of female reproductive organs, maintaining female secondary sexual characteristics, and ensuring vitality and youthfulness. They also maintain the maturity and health of various body systems [45]. Progesterone primarily prepares the endometrium for implantation following estrogen action, while androgens play roles in follicular atresia and the enhancement of libido.

As early as 6 weeks into fetal development, primordial germ cells migrate from the yolk sac to the genital ridge. At birth, a newborn’s ovaries contain approximately 100,000 to 500,000 primordial follicles, but 99.9% of these follicles will undergo atresia [46]. At the start of each menstrual cycle, multiple follicles begin to develop, but typically only one or two follicles mature and ovulate, while the others undergo atresia. Over a woman’s lifetime, approximately 400+ follicles will mature and ovulate, with the remaining immature primordial follicles forming the basis of the ovarian reserve.

#### Key components and functions of ovarian tissue

Follicles, as the core functional units of the ovaries, comprise oocytes, granulosa cells, and theca cells [47]. Follicles expressing high levels of FSH (Follicle Stimulating Hormone) receptors are preferentially activated, giving rise to primary follicles surrounded by cuboidal granulosa cells. As follicles develop, granulosa cells proliferate and form several layers of envelopment, gradually giving rise to the outermost layer known as the follicular membrane cells [48, 49]. The oocyte secretes glycoproteins, contributing to the formation of the zona pellucida. As fluid accumulates in the follicular antrum, the follicle transitions into an antral follicle. Under the surge of luteinizing hormone (LH), oocytes complete the first meiotic division, and following ovulation, they accomplish the second meiotic division [50]. Additionally, AMH (Anti-Müllerian Hormone) is secreted by the granulosa cells of antral and pre-antral follicles and serves as a key indicator of ovarian reserve. As follicles undergo damage or depletion, AMH levels decrease, eventually approaching zero after menopause.

The secretion of ovarian hormones is intricately regulated by the coordinated and complex hypothalamic–pituitary–ovarian axis [51]. Influenced by higher brain centers, the hypothalamus releases Gonadotropin-Releasing Hormone, which governs the release of LH and FSH from the pituitary gland. These hormones traverse the bloodstream to the ovaries, regulating gonadal development and sex hormone secretion [52]. Under the influence of LH and FSH, the ovaries undergo cyclical changes in follicular maturation and ovarian hormone secretion, impacting the state of the endometrium and influencing fertilization and implantation. Ovarian hormones also exert a negative feedback effect on the hypothalamus and pituitary gland, thereby modulating the cyclical secretion of ovarian hormones associated with LH and FSH. During the ovulation process, a positive feedback regulation between LH and estrogen secretion promotes the expulsion of the follicle [53, 54].

#### Microenvironment with ovarian tissue

The ovarian stroma, encompassing tissues beyond the follicles, constitutes a diverse array of structures and cells, establishing a stable and conducive environment for follicular development and hormone secretion [55]. Within this stroma, various immune cells, including macrophages and lymphocytes, assume roles in defense and signal transmission. The vascular system plays a pivotal role in the transportation of oxygen, nutrients, and hormones, contributing to the overall functionality of the ovaries. Neurons and neuroglial cells emerge as essential components in hormone regulation and vascular constriction.

Furthermore, lymphatic vessels assume significance in maintaining fluid balance and participating in hormone-induced tissue remodeling [56]. While the ovarian surface epithelium is implicated in tumor genesis, it also contributes to the post-ovulation repair of the ovary. The robust structure of the tunica albuginea serves as a protective barrier against ovarian damage. Ovarian hilum cells, stem cells, and fibroblast-like cells play indispensable roles in the formation of the ovarian stroma.

Cells within the ovarian stroma are prolific in secreting an array of growth factors, cytokines, and other signaling molecules [57]. These substances intricately regulate follicular growth and development; for example, growth factors actively promote the maturation of oocytes within follicles and foster the overall growth of the follicles. This collaborative network of components functions harmoniously to sustain the optimal functionality and health of the ovaries.

### Significance of ovary preservation

**Fig. 3:**
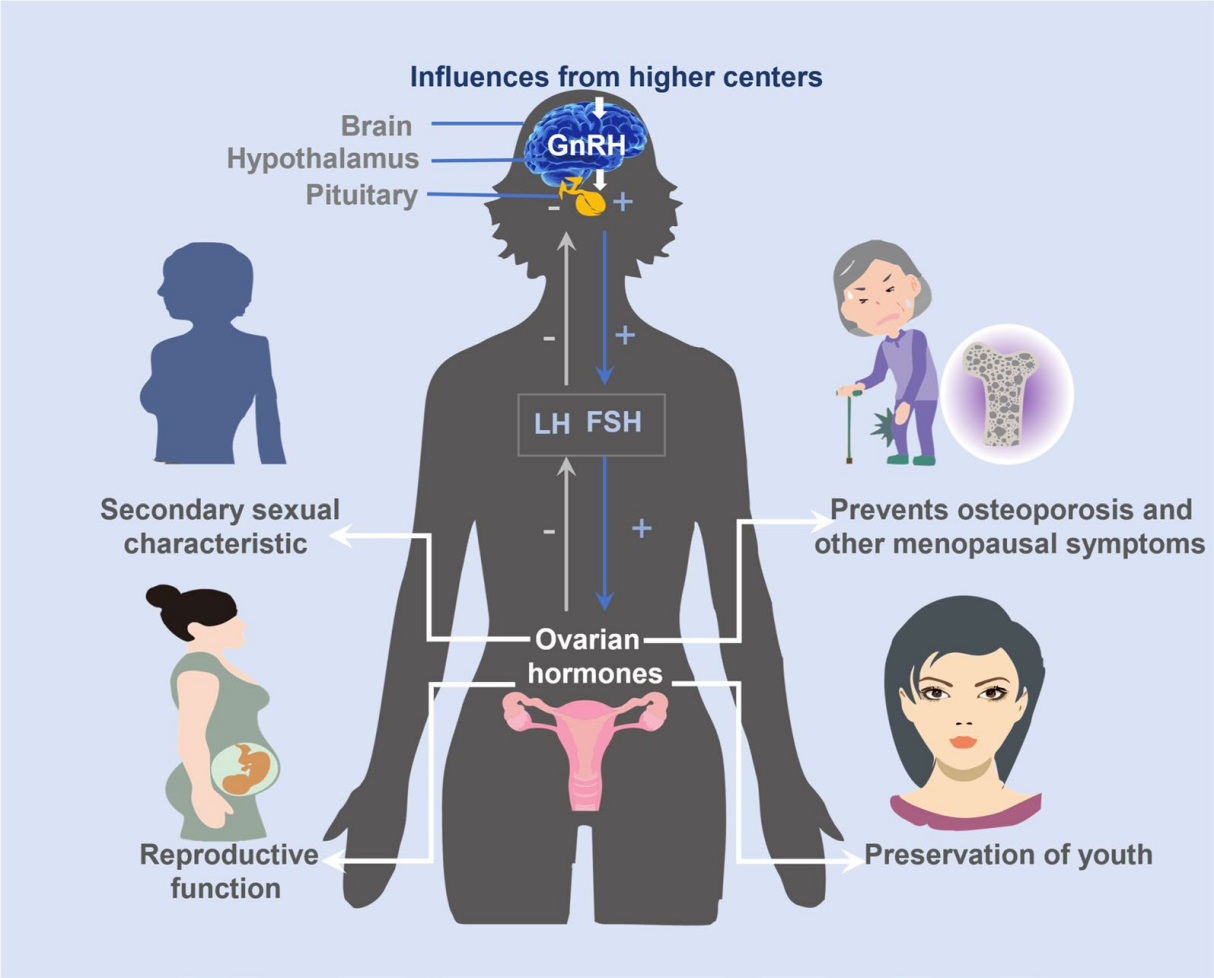
Major applications of ovarian hormones in female physiology and health. The secretion of ovarian hormones is mainly affected by the hypothalamic–pituitary–ovarian axis, and the secreted ovarian hormones such as estrogen, progesterone, androgen, and other hormones and growth factors play an important role in promoting the development of secondary sexual characteristics, maintaining fertility, maintaining youthful state, and preventing perimenopausal symptoms such as osteoporosis

The ovaries, beyond orchestrating the development and maturation of eggs, intricately govern the synthesis and release of hormones (Fig. 3). Presently, factors contributing to ovarian damage are diverse, encompassing age, surgical interventions, radiation therapy, chemotherapy, immune disorders, and lifestyle choices, among others [58]. Regrettably, once the ovaries incur damage, the repercussions are irreversible, and the regeneration of damaged eggs remains beyond reach.

In the era of advancing technology and elevated living standards, there has been a profound impact on our reproductive health and overall well-being. This impact is particularly pronounced in young cancer patients, where radiotherapy and chemotherapy [59] can result in the permanent loss of fertility, accompanied by health issues arising from ovarian dysfunction.

Consequently, safeguarding the ovaries assumes paramount significance. In the face of treatments that may affect fertility, such as radiotherapy and chemotherapy, the standard methods for preserving fertility include preservation of mature eggs, embryo preservation, and cryopreservation of ovarian tissue [60]. Freezing of mature eggs and embryos is suitable for married women, who can obtain mature eggs and embryos for cryopreservation by means of medical ovulation induction and in vitro fertilization. For unmarried sexually mature women, egg freezing is an option to preserve fertility. However, for patients who require immediate chemoradiotherapy and for prepubertal women, freezing and transplantation of ovarian tissue becomes the only option [61]. Because the eggs of prepubertal women are not yet mature enough to be frozen for ovulation induction, ovarian tissue freezing can not only preserve potential oocytes, but also protect the overall function of the ovary. In addition, in patients who need to start cancer treatment as soon as possible, performing procedures such as ovarian hyperstimulation and oocyte retrieval may delay treatment time and increase the risk of tumor spread and complications [62]. Therefore, for patients who cannot delay treatment, ovarian tissue freezing and transplantation have also become their only option [63].

#### Reproductive capacity maintenance

The primary role of the ovaries is to facilitate the development and maturation of ova (eggs). However, various factors, including benign and malignant tumors, infections, environmental pollution, and unhealthy lifestyle habits, significantly influence the normal functioning of the ovaries. This disruption can result in a decline in female fertility, ultimately leading to infertility. Recent reports reveal that the global incidence of infertility has surged to a staggering 15%. Female factors contribute to 45% to 50% of these cases [64], with ovulation disorders being a major contributing factor.

As the demographic affected by cancer skews younger, the impact of cancer has become an overwhelming burden on individuals’ lives. While medical advancements have preserved lives through surgery and chemotherapy, these treatments concurrently strip individuals of the ability to conceive future generations. Such a dual blow is a challenge that many find arduous to endure [65]. Beyond cancer patients, environmental pollution and the rapid modern lifestyle have also profoundly affected the reproductive capacity of the current generation of young people [66]. While technological advancements have brought numerous benefits, they have also generated several adverse effects on the transmission of life.

Therefore, safeguarding the ovaries is tantamount to protecting the ‘roots’ of humanity and securing its future.

#### Impact on female physiological equilibrium and well-being

The role of the ovaries extends beyond the production of eggs, encompassing the synthesis and release of hormones crucial for the physiological development of females. For prepubescent girls undergoing cancer treatment, while surgery and chemotherapy save lives, the absence of ovarian hormone stimulation may impede the normal development of secondary sexual characteristics. The maturation of the uterus could also be compromised. As their peers experience cyclic ovulation and menstruation under the regulation of the hypothalamic–pituitary–ovarian axis, the psychological well-being of these prepubescent cancer survivors becomes a significant concern. They may grapple with self-doubt, depression, and feelings of differentiation from their peers, leading to social isolation, negativity, and even resentment toward the world, potentially losing the courage to live [67].

Moreover, patients facing benign conditions requiring chemotherapy, such as systemic lupus erythematosus, multiple sclerosis, and aplastic anemia, also confront physiological and psychological challenges stemming from ovarian damage [68]. Concurrently, seemingly healthy young women encounter premature ovarian insufficiency, experiencing perimenopausal symptoms like vaginal dryness, decreased libido, irritability, and insomnia. These situations underscore the substantial impact of ovarian health on a woman’s overall well-being, emphasizing the necessity for increased attention and research in this domain.

Therefore, the ovaries not only play a pivotal role in maintaining the physiological balance of women but also influence the generation of happiness on an emotional and psychological level [69]. For individuals grappling with ovarian dysfunction, OTC-T emerge as a crucial therapeutic approach. This method aids in restoring and maintaining physical health while enhancing individual happiness and quality of life on a psychological and emotional level. Through this approach, it becomes possible to effectively promote the overall well-being of patients, encompassing both physiological health and emotional and psychological well-being.

#### Aging and ovary-related disorders

The passage of time in a woman’s life is intricately linked with the depletion of ovarian follicles, a phenomenon commonly known as aging. Aging unfolds through various facets, including diminished skin elasticity, the emergence of wrinkles, alterations in pigmentation, hair loss, and the onset of osteoporosis. Among these, the most pivotal factor is the depletion of ovarian follicles [70]. Numerous physiological and pathological factors can instigate irregular ovarian hormone secretion, thereby expediting the aging process. Ovarian aging can be categorized into two distinct types: physiological aging and premature ovarian insufficiency [71]. In physiological aging, as a woman progresses in age, her ovarian follicles gradually diminish, leading to a decline and degeneration of ovarian function. On the other hand, premature ovarian insufficiency typically afflicts women under the age of 40, characterized by the premature depletion of ovarian function.

Precocious ovarian insufficiency can arise from diverse factors, including genetic disorders like Turner syndrome (also recognized as congenital ovarian dysgenesis syndrome), marked by the partial or complete loss of one X chromosome (most commonly 45, X). This condition is characterized by significantly diminished or absent ovarian volume and extremely low or absent ovarian reserves [72]. Furthermore, benign ovarian conditions such as chocolate cysts and polycystic ovary syndrome may inadvertently inflict damage to some normal ovarian tissue during procedures like ovarian tumor removal or ovarian drilling, thereby hastening the aging process [73]. Ovarian inflammation, encompassing conditions like salpingitis and pelvic inflammatory disease, can also induce ovarian damage by impacting the blood supply around the ovaries. Lastly, surgical interventions for benign and malignant ovarian tumors, along with the administration of chemotherapy drugs, frequently result in iatrogenic reductions in ovarian reserves. All these aforementioned conditions may act as triggers for the initiation of the aging process. For individuals grappling with these conditions, OTC-T may emerge as an efficacious avenue for retarding the aging process.

### Challenges of OTC-T

Ovarian preservation holds profound significance; however, determining the optimal approach is crucial. Currently, two research-intensive methods are under consideration. The first involves whole ovary preservation, wherein the patient’s ovary is stored intact in liquid nitrogen. When puberty initiation or a desire for fertility arises, the ovary is vascularized and anastomosed to restore ovarian function. Although this method facilitates the timely restoration of blood supply due to vascular reconstruction, it poses challenges in preserving a large number of intact ovaries. Uniform and complete penetration of cryoprotectants throughout the entire ovary proves to be challenging, and potential issues such as anastomotic stenosis and thrombosis after the operation remain fully recognized, given the limited number of successful cases [74].

**Fig. 4:**
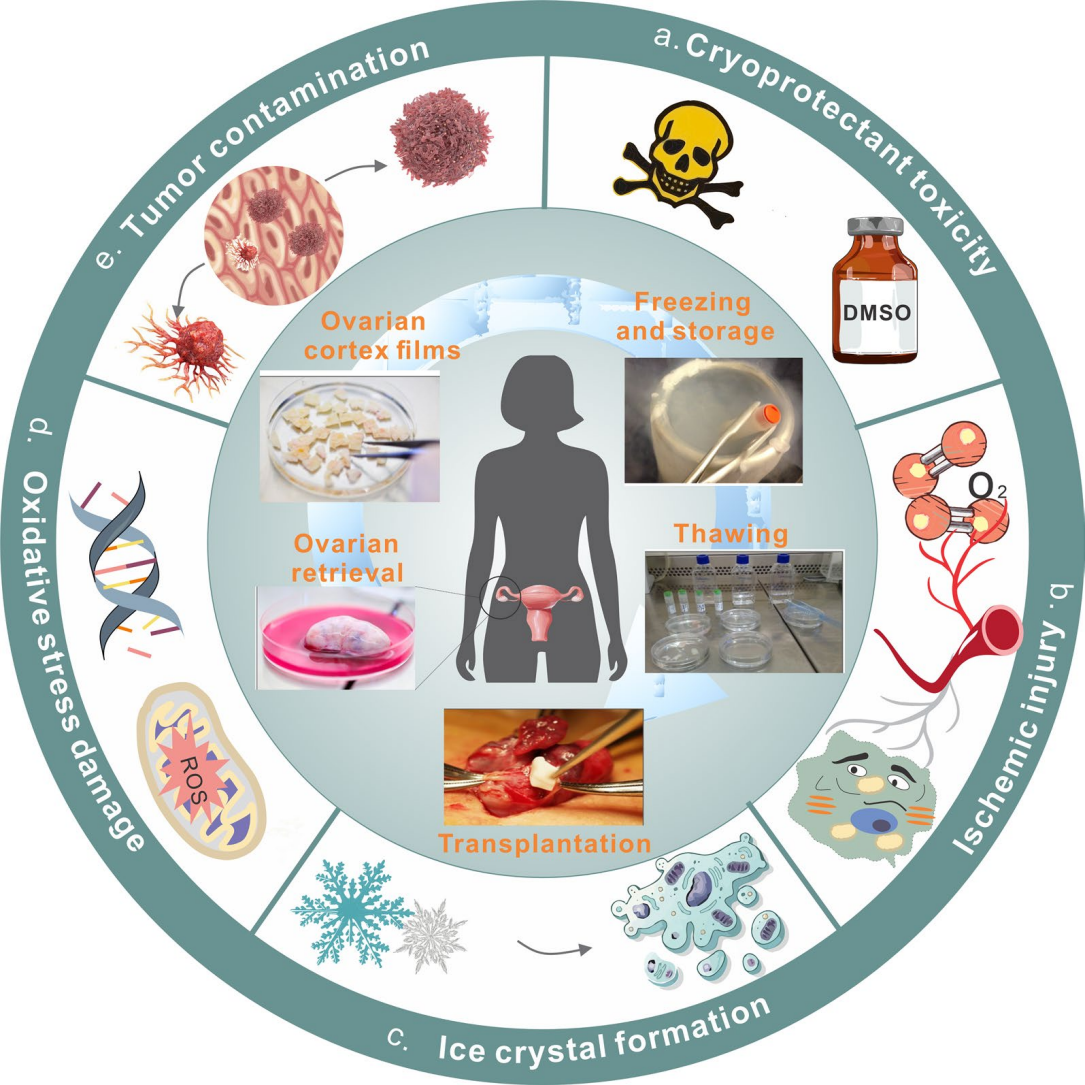
Ovarian tissue cryopreservation-transplantation process and challenges. Ovarian tissue cryopreservation-transplantation process: the ovaries are removed, the medulla is removed, the cortex is prepared, and the cortex tablets are frozen and thawed for orthotopic or ectopic transplantation [76]. Challenges in this process include: **a** toxicity of cryoprotectants. **b** Ischemic damage caused by early lack of vascular support. **c** Fatal damage to cells caused by ice crystals. **d** Oxidative stress damage after revascularization. **e** Risk of transplant reinfection caused by tumor contamination

The second method, OTC-T, involves preserving only the cortical part of the ovary for subsequent transplantation. OTC-T offers advantages such as a smaller tissue mass for convenient preservation and improved cryoprotectant penetration, minimizing cryoinjury. Additionally, multiple transplants are feasible, mitigating the risk of losing the chance for transplantation after a single failure. However, OTC-T faces challenges such as a lack of vascular support, leading to early ischemia and hypoxia causing substantial follicle loss. Moreover, the reintroduction of blood transfusion may result in the overproduction of reactive oxygen species, leading to oxidative stress injury. These challenges underscore the need for ongoing research and innovative solutions in the field of OTC-T (Fig. 4) [75].

#### Tissue damage in cryopreservation

OTC-T is recognized worldwide as a fertility preservation technique, but the preservation method is still controversial, mainly including vitrification freezing, programmed freezing, and ultra-rapid freezing [77]. vitrification freezing refers to the rapid freezing of ovarian tissues by exposing the ovarian tissues to high concentrations of cryoprotectant in liquid nitrogen, to make the cells vitrify rather than freeze, thus preventing the formation of ice crystals from causing cellular damage, but the reality, especially in the rewarming process still cannot completely inhibit ice crystal formation. Programmed freezing, also known as slow freezing [78], refers to the gradual cooling of tissues placed on a device in liquid nitrogen, which has the advantages of simplicity, high efficiency, does not require the use of large quantities of cryoprotectant, and can be programmed to control the rate of freezing, and the majority of live births reported so far are from programmed freezing, which is also likely to be related to the earlier application of programmed freezing. Ultra-rapid freezing is a freezing technique between vitrification and slow freezing that allows the sample vials to be placed directly into liquid nitrogen. It is faster than programmed freezing but also does not involve the application of a programmed machine while using a lower concentration of cryoprotectants (CPA) than vitrification freezing [79].Currently, ultra-rapid freezing is mainly used for embryo and oocyte freezing, and its application in OTC has rarely been reported.

As of present, a consensus on the optimal cryopreservation method for OTC-T remains elusive. In 2021, Marisa Kometas et al. undertook a comprehensive review encompassing clinical and experimental studies from January 2012 to June 2020 [80]. They discovered that only nine biochemical studies directly compared the application of these two freezing methods in human ovarian tissue, with the majority of clinical studies leaning towards slow freezing. While most studies did not detect significant disparities in follicle morphology and distribution between these methods, this finding necessitates consideration within the realm of methodological variability.

In 2022, Andreas Schallmoser analyzed ovarian tissue from 30 patients subjected to both slow freezing and vitrification, revealing no significant differences in follicle viability among tissue samples [81]. Subsequently, in 2023, Supriya Behl et al. conducted a systematic review and meta-analysis covering literature from January 2006 to January 2022. Their analysis unveiled a notably higher proportion of intact stromal cells in vitrified tissue compared to tissue subjected to slow freezing [82]. However, no significant differences were discerned between the two deep cryopreservation methods concerning intact primordial follicle proportion, DNA fragmentation proportion, or average primordial follicle density.

However, regardless of the freezing method, all of them face the risk of damage to cells and tissues caused by ice crystal re-formation and the toxic damage of CPA such as DMSO [83]. Therefore, there is an urgent need for effective methods to reduce ice crystal formation and to perform rapid and uniform rewarming to improve cryopreservation efficiency.

#### Functional impairments in transplantation

In addition to damage during freezing and rewarming, ovarian tissue also faces significant challenges after transplantation. Although ovarian tissue transplantation has been shown to restore endocrine function and fertility in patients, frozen and thawed human ovarian tissues experience significant follicular loss in the early post-transplantation period due to exposure to hypoxia [84], ischemia and hypoxia are the main causes of follicular loss after transplantation, and reoxygenation takes about 4–5 days, and increased reactive oxygen species after revascularization (ROS) can cause oxidative stress to further exacerbate tissue injury, and although strategies using growth factors [85, 86], various hormones, and antioxidants [87, 88] have been shown to be effective in promoting angiogenesis and reducing oxidative stress, it remains a challenging challenge to more efficiently promote and monitor grafted tissue survival and to reduce oxidative stress injury.

#### Current methodological limitations

Among the strategies to reduce ice crystal formation and growth, anti-apoptotic agents such as S1P (Sphingosine 1-phosphate) [89], AMH [90], Slush Nitrogen (SN) [91], etc. are being investigated, and the most commonly used method is the use of CPAs, which are mainly classified into two types: intracellular and extracellular.

The use of non-permeating CPAs such as sucrose, a single polymer (Supercool X-1000), or combinations of polymers (Supercool X-1000 and PVP K-12) [92], as well as the combined use of permeating and non-permeating CPAs, may potentially enhance the efficiency of vitrification for ovarian tissue [93]. However, the majority of cryoprotectants are often not able to penetrate the cells, resulting in a limited effect on reducing intracellular ice crystal formation. Limited, at the same time, some CPAs also have toxicity problems, which may cause damage to the cells, so how to promote non-toxic cryoprotectants into the cells is a problem that needs special attention. In addition, the success of rewarming also depends on the speed of warming, and uneven rewarming can also cause ice crystal re-formation, which can also cause fatal damage to cells, so rapid and uniform rewarming technology is needed to meet this challenge, and laser-assisted thawing technology has been widely studied, and it is expected to bring new hope for improving the outcome of OTC. Strategies to promote angiogenesis and reduce oxidative stress injury have also been extensively studied, such as growth factors, various hormones, antioxidants, etc., such as S1P has been shown to reduce ischemic reperfusion injury and promote neo-angiogenesis in ovarian grafts [94]. However, it cannot promote follicular proliferation or prevent DNA damage during freezing and thawing. Therefore, there is an urgent need for a method that can rapidly and effectively promote angiogenesis while also safely and noninvasively reducing excess reactive oxygen species in tissues and preventing oxidative stress.

There is a risk of malignant tumor cell contamination during OTC-T. Studies have revealed instances of post-transplant tumor reinfection, which poses a serious threat to patient health. This finding points out that special attention needs to be paid to tissue contamination due to ovarian-origin and blood-origin malignancies [95, 96]. Currently, one approach to eliminate rhabdomyosarcoma and leukemia cells that have metastasized to the ovarian tissue is to use YAP/TAZ inhibitors prior to auto-transplantation [97], but this approach may cause damage to normal cells. Therefore, exploring non-invasive and safer means of elimination has become the key to solving this challenge.

To address these challenges, the development of innovative solutions has become an urgent necessity. In recent years, the application of nanoregulators in tissue-cell therapy has begun to show its potential. This study reviews the performance of nanoregulators in solving the challenges related to OTC-T and provides insights for developing new ideas by combining the principles of their application in cryo-transplantation of various organs and tissues. We believe that with the help of nanotechnology, the field of OTC-T will see new developments in the near future, just as nanotechnology has shown great potential in other fields.

## Nanoregulator design for dynamic restoration

Designing a nanoregulator for dynamic restoration, particularly in the context of ovarian cryopreservation and transplantation, involves intricate considerations. The primary aim is to create a regulatory system that can adapt dynamically to the unique challenges presented by the preservation and transplantation of ovarian tissues. This specialized nanoregulator must be equipped with responsive elements capable of sensing and responding to the specific conditions associated with the cryopreservation process. Its design should encompass features that facilitate real-time adjustments, ensuring optimal functionality during the transplantation phase. In the realm of ovarian cryopreservation and transplantation, the nanoregulator becomes a crucial tool, offering precision and adaptability for achieving successful outcomes in fertility preservation. Through innovative design and tailored engineering, nanoregulators contribute to advancing the field of dynamic restoration, especially in the intricate procedures of ovarian cryopreservation and subsequent transplantation.

### Essential background for nanoregulator design

To gain a profound insight into the intricate interactions among substances at the microscopic level, as well as the precise regulation and manipulation of chemical reactions on the nanoscale, the concept of a nanoregulator has been introduced [98]. This innovative device, functioning at atomic, nano, or sub-micron scales, serves as a sophisticated reactor meticulously crafted to address practical applications. Its primary objective is to overcome challenges inherent in traditional scales, such as catalytic efficiency and reaction rates.

The nanoregulator harnesses the extraordinary physical and chemical properties of nanoregulators, strategically exploiting them to optimize and enhance the efficiency and selectivity of chemical reactions. This optimization involves meticulous adjustments to the structure, composition, and surface properties, unlocking the full potential of nanoregulators. The distinctive internal region of the nanoregulator creates an advantageous environment for executing specific chemical reactions. By confining the movement of atoms and other reactants, it establishes reaction conditions distinct from the surrounding environment. Consequently, the kinetics and overall process undergo transformation when chemical reactions occur within the confined space of nano-size and micrometer volume.

This design concept mirrors the organization of cells in living organisms, providing segregated yet complete spaces that facilitate diverse reactions. As nanoscience and nanotechnology advance continuously, nanoregulators have exhibited promising outcomes across various domains, including biomedicine and energy conversion [99, 100].

### Classification and mechanisms of the nanoregulator

Nanoregulators manifest in both natural and synthetic forms, with natural variants encompassing esteemed entities like biological macromolecules, protein cages, and viruses. Meanwhile, synthetic counterparts span a diverse array, embracing molecules, polymers (ranging from polymers to polymer micelles and hydrogels), as well as single-molecule nanoregulators like microemulsions, shell-nucleus nanostructures, porous solids, and nanotubes. These sophisticated entities predominantly serve catalytic functions, engage in drug delivery at the nano-level, and act as biosensors [101–103].

The intricate mechanisms underlying nanoregulators are intricately tied to the quantum effects exhibited by the materials, thereby altering their electronic structures and influencing processes such as photoelectrocatalysis. Furthermore, surface effects come into play, leveraging substantial specific surface areas to augment active sites and enhance catalytic efficiency. In addition, nanoregulators exhibit remarkable sensitivity, displaying a heightened responsiveness to local stimuli, thereby elevating their efficacy and impact (Table 1).

**Table 1:** Classification, characteristics, and clinical application of common nanoregulators in the biomedical field

Materials	Representative	Characteristics	Application
Organic	• Polylactidecoglycolide (PLGA) • Polylactic acid (PLA) • Polyamidoamine (PAMAM) • Chitosan • Liposomes • Nanoemulsions	• Possesses good biocompatibility and biodegradability • Simple physicochemical properties, making surface modification easier • Offers high encapsulation efficiency and prolonged circulation time in the body • Demonstrates relatively low toxicity or non-toxic properties • Suitable for various drug delivery routes, although thermodynamically unstable when exceeding 500 nm in size	• Drug carriers for controlled and sustained release • Construction of artificial tissues and organs • Gene therapy • Nanorobot • Delivery of vaccines and anticancer agents
Inorganic	• Metal • Metal oxide	• Unique electrical, optical, and magnetic properties • Inertness, stability, and ease of functionalization • Unique surface characteristics (such as small pore size, large variation of surface charge and density, a large ratio of surface area to volume, diverse shape, and structure) • Toxicity and poor biocompatibility • Metal oxides have high reactivity	• Biosensor and diagnostic imaging (e.g. MRI) • Magnetic transfection • Hyperthermia • Antibacterial and antiviral therapy • Targeted therapies • Cell isolation • Vaccine development • Optimization of drug and gene delivery • Regenerative medicine • Stem cell growth and differentiation • Nucleic acid amplification
Ceramics nanoregulators	• Titanium oxide (TiO_2_) • Hydroxyapatite (HA) • Alumina (Al_2_O_3_) Silica (SiO_2_)	• Non-metallic solid with photocatalysis, photodegradation, imaging, and cytotoxicity	• Photodynamic therapy • Drug activity can automatically release drugs
Carbon-based nanoregulators	• Quantum dots • Fullerenes • Carbon nanotubes • Graphene-and-its derivatives	• Extremely large surface area, chemical purity and free electrons, Photoluminescence properties, Excellent biocompatibility, and photobleaching durability	• Drug delivery • Biomolecule sensing • Cancer treatment • Imaging • Targeted drug delivery

#### Size and composition variations

The adjustments in the size and composition of nanoregulators are primarily tailored to suit specific applications, thereby showcasing a vast spectrum of potential in fields such as biomedicine and materials science. This versatility is realized through meticulous adjustments to the structure, material, and preparation methods of the nanoregulator. Diverse sizes and compositions yield an array of nanoregulators, predominantly classified as inorganic nanoparticles, lipid nanoparticles, bionic nanoparticles, hydrogel nanoparticles, and more. Such precision in customization underscores the adaptability and expansive utility of nanoregulators across varied disciplines.

##### Inorganic nanoparticles

Inorganic nanoparticles, encompassing carbon-based nanoregulators, metal nanoparticles, magnetic oxide nanoparticles, and more, exhibit a distinctive set of properties owing to their nanoscale dimensions. The remarkable specific surface area at this scale imparts these nanoparticles with entirely different attributes compared to their macroscopic counterparts. As a result, they possess unparalleled physical, chemical, mechanical, and biological characteristics. The expansive specific surface area significantly augments the contact area of the reaction and active sites, bestowing inorganic nanoparticles with a pronounced advantage in applications such as adsorption and catalysis [104].

These inorganic nanoparticles find prominent utility as biosensors or diagnostic imaging tools, contribute to antibacterial and antiviral therapies, facilitate thermotherapy, and serve as integral components in drug delivery optimization by functioning as carriers [105–109]. The strong variability in the size, shape, and structure of inorganic nanoregulators, coupled with their unique optical, electrical, and magnetic properties, underscores their versatility. However, it is imperative to acknowledge the potential toxicity associated with inorganic nanoregulators, a factor that necessitates judicious consideration in their application. Despite their extraordinary attributes, the presence of toxicity underscores the importance of prudent handling and assessment in the realm of nanoparticle manufacturing [110, 111]. In the realm of reproductive toxicity, particularly the issues such as disturbances in germ cell differentiation and impacts on embryonic development, have garnered widespread attention [112, 113]. Therefore, while leveraging the unique advantages of inorganic nanoparticles, it is imperative to balance these with their potential negative issues, such as toxicity.

##### Lipid nanoparticles

Lipid nanoparticles (LNPs) are nanoparticles composed of lipids with a homogeneous lipid core, which are scaled at the nanoscale (typically between tens and hundreds of nanometers), significantly smaller than human cells (~ 7 μm), can effectively cross cell membranes and other biological barriers, and are capable of encapsulating a variety of drugs, including small-molecule drugs, proteins, and RNA. It also prevents enzymatic degradation of drugs and also reduces adverse drug reactions [114, 115]. By modifying their surface properties [116–118], LNPs can be designed to target specific tissues or cell types, thereby improving therapeutic relevance and efficiency. They are mainly used as controlled- and sustained-release drug carriers [119, 120], gene therapy [121, 122], artificial tissue and organ construction [123], vaccine development [124], and anticancer drug delivery [125], and have the advantages of good biocompatibility and biodegradability, high encapsulation efficiency, long circulation time, nontoxicity or relatively low toxicity, and slow clearance by body immune cells [126, 127].

##### Bionic nanoparticles

Bionic nanoparticles are meticulously crafted through the emulation of natural processes or structures inherent in living organisms. Encircled by a biomimetic encapsulating layer reminiscent of a cell membrane, these nanoparticles acquire enhanced biocompatibility and prolonged retention time within the bloodstream [128, 129]. This characteristic extends their efficacy within the organism, concurrently evading immune system clearance and proteolysis.

The adjustment of size, shape, and surface charge bestows upon these nanoparticles the capacity to replicate cellular and viral functions. They serve as inducers, attracting and neutralizing toxins, thereby mitigating damage to healthy cells. Moreover, these nanoparticles exhibit the capability to mimic specific biomolecular functions, such as molecular recognition specificity and targeted properties against cancer [130, 131]. This attribute has spurred extensive research in the realm of targeted cancer therapy.

Notably, these nanoparticles are frequently synthesized through environmentally conscious, green methods that eschew the use of hazardous substances. This dual emphasis on functionality and ecological responsibility renders them versatile and eco-friendly nanoregulators [132, 133].

##### Hydrogel nanoparticles

Hydrogel nanoparticles represent nanoscale particles characterized by cross-linked polymer networks with remarkable hygroscopic nature, proficiently absorbing and retaining substantial volumes of water without dissolving. Crafted from biocompatible materials, these particles boast excellent biocompatibility [134]. Moreover, they exhibit tunability and responsiveness, adapting to local environmental conditions such as temperature and pH.

Presently, hydrogel nanoparticles find ubiquitous applications across various domains, including but not limited to the following [135]: augmenting the stability and bioavailability of drugs to enhance the efficiency and effectiveness of drug delivery. They play a pivotal role in cell culture and tissue engineering, replicating the in vivo environment to foster cell growth and tissue regeneration. Additionally, hydrogel nanoparticles serve as adept biomarker detection and imaging tools, offering heightened sensitivity and specificity. Furthermore, they can be meticulously engineered to target specific cells or tissues, enabling precision therapy with minimized side effects.

These remarkable properties position hydrogel nanoparticles as promising contenders across a diverse spectrum of applications in medicine, biology, and materials science. They offer innovative solutions to tackle an array of biomedical and drug delivery challenges, showcasing their potential for transformative contributions in these fields.

#### Targeting ovarian cells and identification

Nanoregulators employ sophisticated mechanisms to target and identify ovarian cells, ushering in a new era of precision in cellular interventions [136]. These cutting-edge agents leverage a nuanced approach, recognizing distinct cell types within the ovarian microenvironment. Granulosa cells, theca cells, and the surrounding mesenchymal vascular endothelial cells become focal points for nanoparticle targeting, creating a tailored strategy for cellular interaction.

The targeting mechanism proves invaluable in monitoring the survival status of ovarian tissue grafts and promoting revascularization within the ovarian tissue [137]. Nanoparticles, endowed with magnetic and thermal conductivity properties, showcase their prowess in mitigating cell damage caused by ice crystal formation during cryopreservation [138]. Magnetic induction heating of magnetic nanoparticles stands out as a novel method in this regard, contributing to the preservation of ovarian cellular integrity.

Furthermore, the customization of specific ligands on the surface of nanoparticles, such as AMH antibodies or small molecules, enables receptor-mediated targeted delivery [137]. This sophisticated approach ensures the precise recognition of receptors on the surface of ovarian cells, exemplified by the recognition of AMH secreted by granulosa cells. This targeted delivery system plays a crucial role in efficiently monitoring the survival status of transplanted ovarian tissue.

In addition to ligand modifications, the fine-tuning of surface properties, including charge, hydrophilicity/hydrophobicity, and surface-modifying molecules [139], enhances the affinity of nanoparticles for specific ovarian cell types. This intricate interplay of properties highlights the advanced nature of nanoregulators in the realm of ovarian cell targeting and identification, offering promising avenues for advancements in reproductive health.

## Role of nanoregulators in ovarian tissue cryopreservation and transplantation

Amidst the relentless march of scientific and technological advancements, significant breakthroughs have unfurled in the utilization of nanoregulators across diverse research domains. Particularly noteworthy are the strides made in areas such as the early diagnosis and treatment of tumors, drug encapsulation and delivery, and tissue engineering.

In recent years, the application of nanoregulators has extended its reach into the realm of female reproduction and health-related fields. Notably, in 2014, Barkalina N et al. provided a comprehensive overview of the use of nanoregulators in reproductive medicine and biology [28]. After that, in 2018 Lloyd-Parry et al. illuminated the application of nanomedicine in s health [26]. The year 2021 witnessed José Roberto Viana Silva et al. delving into the advantages and challenges of nanoregulators in human-assisted reproductive technology [27].

The widespread integration of nanoregulators has proven to be a source of great encouragement. Naturally, one cannot help but contemplate whether the unique advantages of nanoregulators could be harnessed to address challenges in OTC-T. Hence, the exploration of nanoregulators in the realm of OTC-T emerges as a captivating area of interest and developmental focus.

### Ice inhibition of nanoregulators

The primary challenge confronting OTC-T lies in the mechanical damage inflicted upon cells and tissues due to the formation of ice crystals during freezing and subsequent rewarming. Hence, it becomes imperative to proficiently mitigate the initiation and expansion of ice crystals. In recent years, nanoregulators have emerged as formidable tools, showcasing their distinctive capabilities in diminishing ice nucleation formation and impeding the growth of ice crystals. In the ensuing discussion, the authors will delve into both the mechanisms employed by nanoregulators to prevent icing and the refinement of strategies for optimizing ice inhibition in the cryopreservation process.

#### Mechanisms for preventing ice formation

The quest for effective cryopreservation in the context of OTC-T necessitates a nuanced exploration of mechanisms aimed at preventing ice formation. The foremost challenge lies in averting mechanical damage inflicted by the crystallization of ice during freezing and subsequent rewarming. Nanoregulators, at the forefront of this endeavor, wield unique mechanisms to curtail ice nucleation and thwart the growth of ice crystals.

At the molecular level, nanoregulators intervene in the intricate dance of water molecules, strategically minimizing their predisposition to form ice crystals. By leveraging their distinct properties, nanoregulators act as guardians against the deleterious effects of ice formation. This sophisticated intervention not only showcases the advanced capabilities of nanoregulators but also opens promising avenues for refining cryopreservation techniques in the intricate landscape of OTC-T.

**Fig. 5:**
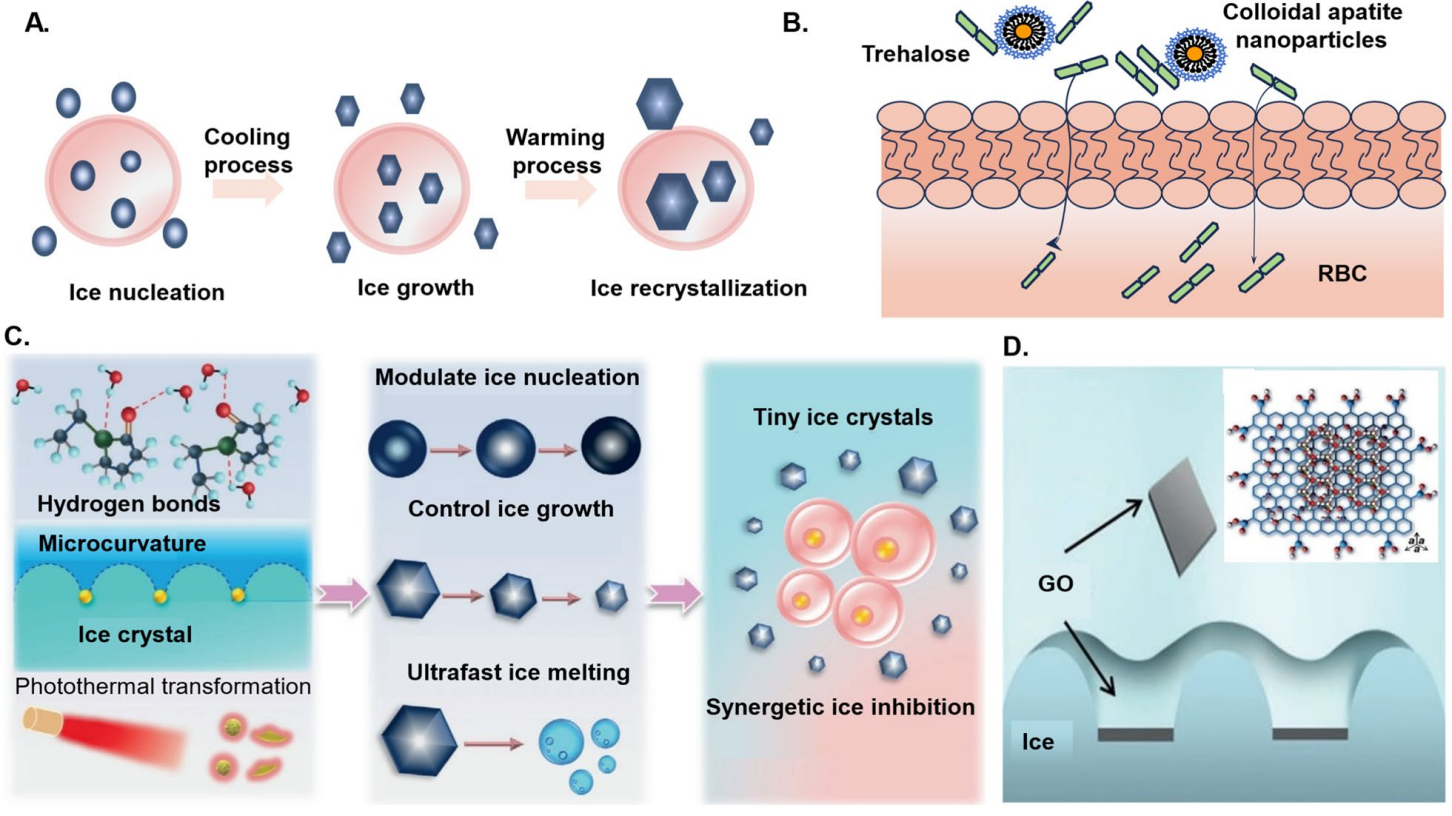
Ice crystal formation process and ice suppression mechanisms of common nanoregulators [141, 142]. **A** Ice crystal formation process. **B** Hydroxyapatite nanoparticles promote the entry of the extracellular cryoprotectant alginate into the cell. **C** WSe2-PVP NPs regulate ice crystal formation and promote ice crystal melting through the synergistic effects of hydrogen bond formation, adsorption inhibition, and photothermal conversion. **D** Graphene oxide inhibits ice crystal growth and recrystallization through the formation of hydrogen bonding with ice crystals, which results in bending of the ice crystal surface

In 2021, Chang et al. [140] unveiled a breakthrough in the realm of nanoregulators, demonstrating their remarkable potential as antifreeze protein-like agents capable of inhibiting ice crystal growth during cell and tissue freezing. Notably, certain nanoregulators exhibit synergistic ice inhibition properties, as depicted in Fig. 5. Take, for instance, WSe2-PVP NPs, which exhibit a multifaceted approach to ice crystal generation and growth inhibition. This involves the formation of hydrogen bonding, modulation of ice crystal microcurvature, and photothermal conversion, collectively enhancing freezing efficiency [141].

Furthermore, select nanoregulators play a pivotal role in facilitating the entry of non-toxic extracellular cryoprotectants into cells. This innovative approach serves to reduce reliance on toxic cryoprotectants, thereby elevating the overall freezing effectiveness while mitigating potential harmful effects. Such strides in leveraging nanoregulators for nuanced cryopreservation techniques underscore their transformative impact on advancing cellular preservation methodologies.

##### Nanoregulators control the formation of ice nuclei and inhibit the growth of ice crystals

Graphene, a cutting-edge carbon-based nanomaterial, manifests as a single layer of tightly stacked carbon atoms arranged in a two-dimensional honeycomb crystal structure. This structure, grounded in the stability of the benzene six-membered ring, represents the fundamental unit in organic materials. Remarkably, with a thickness of only one carbon atom, graphene’s single atomic layer structure allows for scalability to tens of micrometers in lateral dimensions.

Graphene oxide (GO), the oxide derivative of graphene, enhances stability in both aqueous solutions and polar solvents, serving as a surfactant at the interface to reduce interfacial energy. The ice suppression mechanism of GO is intricately linked to its honeycomb hexagonal scaffolds, aligning oxide groups on the basal planes with ice crystals. This alignment fosters increased hydrogen bonding with ice crystals, inducing surface bending, and effectively inhibiting crystal growth and recrystallization [142] (Fig. 5).

In 2017, Geng et al. [143] demonstrated the remarkable impact of incorporating a mere 0.01 wt% of GO into the culture medium, significantly improving the motility of equine spermatozoa. This underscored GO’s prowess in inhibiting ice crystal growth and recrystallization. In a recent study by Fayazi et al. [144], graphene oxide nanoparticles were integrated into the vitrification freezing process of mouse blastocysts as a pioneering nanocryoprotective agent. This innovative approach, replacing sucrose with graphene oxide, yielded comparable re-expansion, hatching, and implantation rates to those of fresh controls. It also demonstrated impressive protective capabilities against various cellular and molecular stresses, positioning GO as a potential vitrification cryoprotectant for embryos.

Furthermore, the study conducted by Zhu et al. in 2019 highlighted the anti-icing properties of nanoregulators (MOF NPs) constructed from zirconium-based metals and organic backbones [145]. This hybrid nanomaterial, mirroring antifreeze proteins, proved invaluable for the cryopreservation of red blood cells, showcasing a significantly higher recovery rate than conventional (toxic) organic solvents. Notably, MOF NPs not only inhibited ice recrystallization but also served as catalysts, expediting ice crystal melting.

**Fig. 6:**
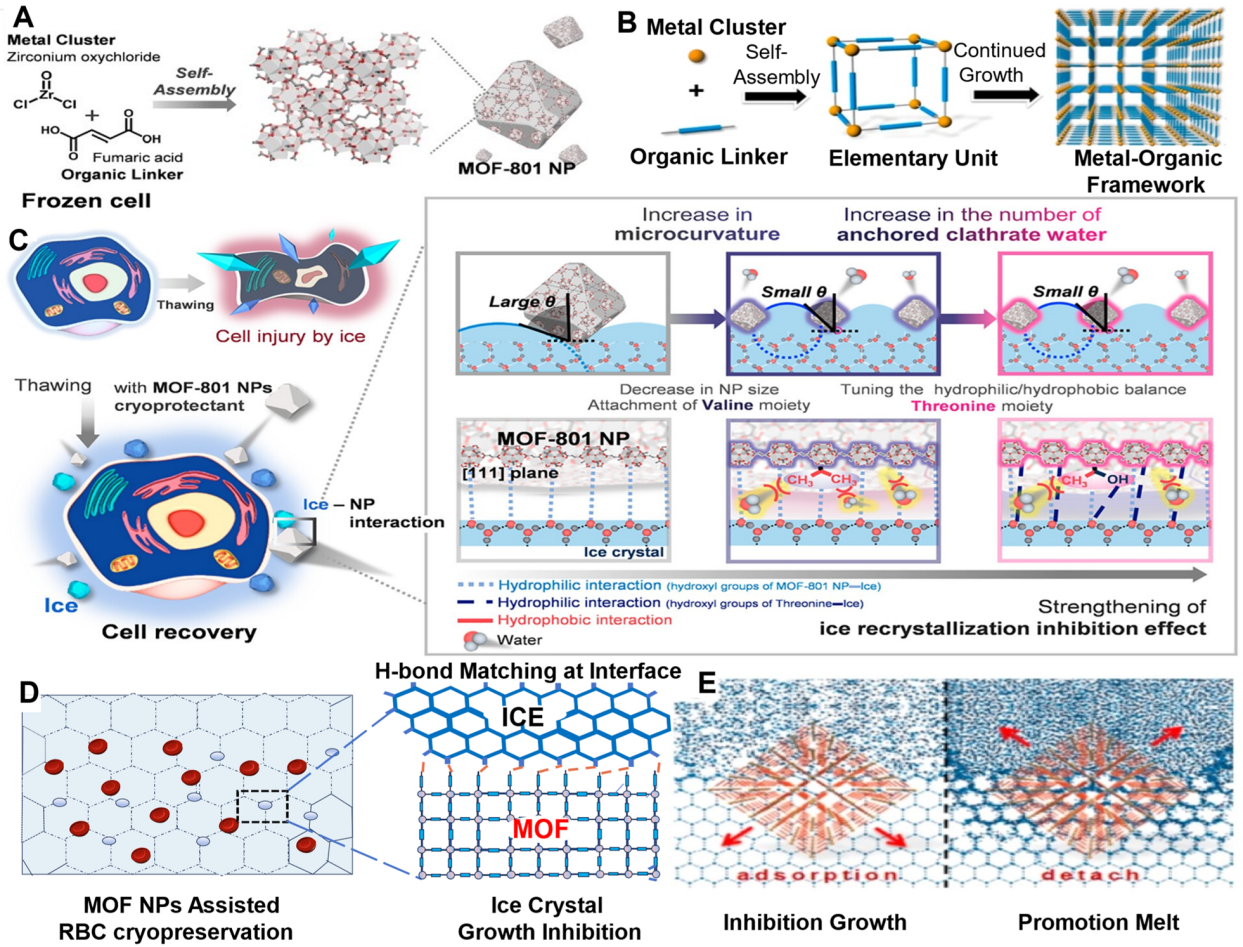
Mechanism of different types of zirconium-based metal and organic frame-based nanoregulators (MOF NPs) inhibiting ice crystal growth [145, 146]. **A** Schematic illustration of the solvothermal synthesis of MOF-801 NPs. **B** The formation process of MOF-808 NPs. **C** MOF-801 NPs as a cryoprotectant for preventing cell injury caused by ice crystal growth during cell freezing and thawing (left). Ice recrystallization inhibition effect of MOF-801 NPs by controlling the NPs size and introducing ice-binding amino acids that affect the micro curvature on the ice surface. The anchored water molecules allow the MOF-801 NPs to adsorb well to certain ice planes (right). **D** MOF-808 NPs form hydrogen bonds with water molecules to inhibit the growth of ice crystals. **E** The equilibrium of adhesion and separation between MOF-808 NPs and the ice surface makes it a “catalyst”, which accelerates the exchange of water molecules at the interface between ice and free water, thus promoting the melting of ice crystals

In 2023, Jeon’s team harnessed MOF NPs as cryoprotectants, capitalizing on their ability to modify organic linkers and control ice surface micro-curvature [146]. This strategic manipulation facilitated the creation of cyclically ordered ice-binding sites, effectively reducing ice crystal formation and advancing the prospects for cellular cryopreservation. These groundbreaking studies collectively underscore the transformative potential of nanoregulators in the realm of cryopreservation, offering innovative solutions for various applications (Fig. 6).

##### Nanoregulators facilitate the penetration of non-toxic cryoprotectants

Nanoregulators play a pivotal role in revolutionizing the cryopreservation landscape by facilitating the penetration of non-toxic cryoprotectants into cellular structures. In the intricate dance of cellular preservation, the introduction of non-toxic extracellular cryoprotectants becomes a strategic maneuver, aiming to reduce reliance on traditional toxic counterparts. This innovative approach not only enhances the overall effectiveness of cryopreservation but also mitigates potential harmful effects associated with toxic cryoprotectants.

The nanoregulators act as facilitators, intricately assisting the entry of non-toxic cryoprotectants into cellular domains. This strategic collaboration between nanoregulators and cryoprotectants not only underscores the advanced capabilities of nanotechnology but also aligns with the contemporary shift towards safer and more sustainable preservation methodologies. By reducing the dependence on toxic agents, nanoregulators contribute to the refinement of cryopreservation techniques, opening new avenues for preserving cellular integrity and function with heightened precision and safety. This transformative synergy reflects the progressive trajectory of nanoregulators in reshaping the landscape of cryopreservation for cellular applications (Table 2).

**Table 2:** Mechanism and application of nanoregulators inhibiting the formation and growth of ice crystals

Nanomaterial	Size	Optimum concentration	Main freezing medium	Experimental object	Affection	Mechanism	Refs.
Graphene oxide	500 nm	1.0 mg/mL	Culture medium	Horse sperm	Significantly improve the motility of horse sperm (from 24.3 to 71.3%)	The shape of the honeycomb hexagonal bracket matches the arrangement of the oxidation groups on the GO substrate with the ice crystal, thus forming a hydrogen bond with the ice crystal, resulting in the bending of the ice crystal surface and inhibiting the growth and recrystallization of the ice crystal	[142, 143]
Graphene oxide	Not mentioned	1.5 mg/mL	VS consisting of 15% Me_2_SO, 15% ethylene glycol (EG), and 0.5 M sucrose	Mouse blastocyst	The re-expansion rate, hatching rate and implantation rate of vitrified frozen embryos were increased, and a series of cellular and molecular stress could be prevented	As mentioned above	[144]
MOF-801 NPs	10 nm	50 μg/mL	Ice-binding proteins	Erythrocyte	Increase red blood cell recovery rate	The formation of ice crystals is reduced by modifying organic connectors and controlling the micro-curvature of the ice surface to provide periodic and orderly ice-binding sites	[145, 146]
Genipin crosslinked Pluronic F127-chitosan NPs	47.7 ± 2.1 nm	0.1–10 mg/mL	Trehalose	Human adipose stem cells	Reduce the injury of adipose stem cells and improve the survival rate	Promote the entry of non-toxic extracellular ice-inhibiting substances into cells and reduce the use of intracellular toxic cryoprotectants	[147]
Hydroxyapatite (HA) NPs	60 nm	0.01%, 0.02%, 0.05%, 0.1% (w/w)	15% (v/v) Me_2_SO, 15% (v/v) EG and 0.5 M sucrose	Porcine oocyte	Improve the survival rate of oocytes	Increase the viscosity of the solution to delay devitrification	[148]
Apatite nanoparticles	22-80 nm	2.25 mg/mL	Trehalose	Erythrocyte	Improve the frozen survival rate of red blood cells	Promote trehalose to permeate into the cell without pore, and significantly improve the anti-freezing ability of the cell	[149]

Moreover, numerous intracellular cryoprotectants exhibit high toxicity, exemplified by substances like DMSO, which, upon entering cells, can induce toxic damage [150]. The subsequent necessity for washing post-thawing introduces an additional risk of cellular damage during this process. Nanotechnology emerges as a transformative force, facilitating the ingress of non-toxic extracellular ice-inhibiting substances into cells. This strategic intervention diminishes the reliance on toxic intracellular cryoprotectants, thereby mitigating cellular damage. Simultaneously, it presents a novel strategy for non-toxic cryoprotection.

Toxic cryoprotectants necessitate innovative approaches, and nanotechnology offers promising solutions. For instance, the biocompatible sugar trehalose can serve as an extracellular ice inhibitor, albeit unable to penetrate the cell membrane [151]. Nanotechnology comes to the rescue by enabling the entry of alginate into cells to mitigate intracellular ice crystal formation. In 2015, Rao’s team [147] utilized genipin-crosslinked Pluronic F127-chitosan nanoparticles (GNPs) to facilitate the entry of alginate into cells, achieving successful freezing of human adipose stem cells using alginate as the sole cryoprotectant.

In the realm of cryopreservation, nanoparticles alter the degassing and recrystallization behavior of cryoprotectant solutions during warming, a phenomenon influenced by solution type, nanoparticle size, and nanoparticle content [152]. In 2015, Zhou’s team [148] applied biocompatible HA nanoparticles to the cryopreservation of porcine oocytes, observing a reduction in ice crystal re-formation during rewarming, leading to improved oocyte survival. While the exact mechanism remains unclear, it is postulated that the increase in solution viscosity by HA nanoparticles contributes to delaying devitrification.

A subsequent study by Stefanic et al. in 2017 [149] delved into the freezing survival of erythrocytes, revealing a significant enhancement upon the addition of apatite nanoparticles to the culture medium. This improvement was primarily attributed to apatite nanoparticles promoting non-porous infiltration of alginate into cells, thereby elevating cellular antifreeze capabilities and reducing freeze-induced damage. These remarkable advancements underscore the transformative impact of nanotechnology in reshaping cryopreservation methodologies for enhanced cellular preservation.

#### Optimization of ice inhibition in cryopreservation

Beyond traditional approaches, contemporary advancements in cryopreservation leverage hydrogels and tissue engineering strategies, encompassing intracellular alginate delivery, cell encapsulation, and innovative bionic structural design [140]. These techniques are increasingly employed for preserving a diverse array of cells, tissues, and organs.

Hydrogels stand out due to their remarkable biocompatibility, thermal reversibility, and tunable mechanical properties, rooted in their distinctive network structure and compositional characteristics [153]. The ice crystal inhibition mechanism of hydrogels primarily involves intricate interactions within the hydrogel network, such as hydrogen bonding, resulting in a reduction of free water proportion [154]. Moreover, the incorporation of charged and hydrophobic groups within hydrogels effectively modulates the icing interface, diminishing ice crystal formation [155].

Cell encapsulation techniques emerge as a strategic solution, minimizing contact with water and mitigating ice crystal formation [156]. Biomimetic structural design strategies capitalize on the temperature-responsive properties of biomimetic materials to alter local ice crystal formation conditions. Additionally, these strategies encompass the design of microstructures on material surfaces to influence the growth pattern of ice crystals and draw inspiration from biological interfaces, such as the skin of Arctic fish, to deter the attachment and growth of ice crystals.

These innovative strategies harness the unique physical and chemical properties of materials to enhance the efficiency and safety of the cryopreservation process. The overarching goal is to minimize damage to cells, tissues, and organs, thereby ushering in a new era of advanced cryopreservation techniques with heightened efficacy and reduced risks.

### Nanoregulators: catalysts for enhanced heat generation

**Fig. 7:**
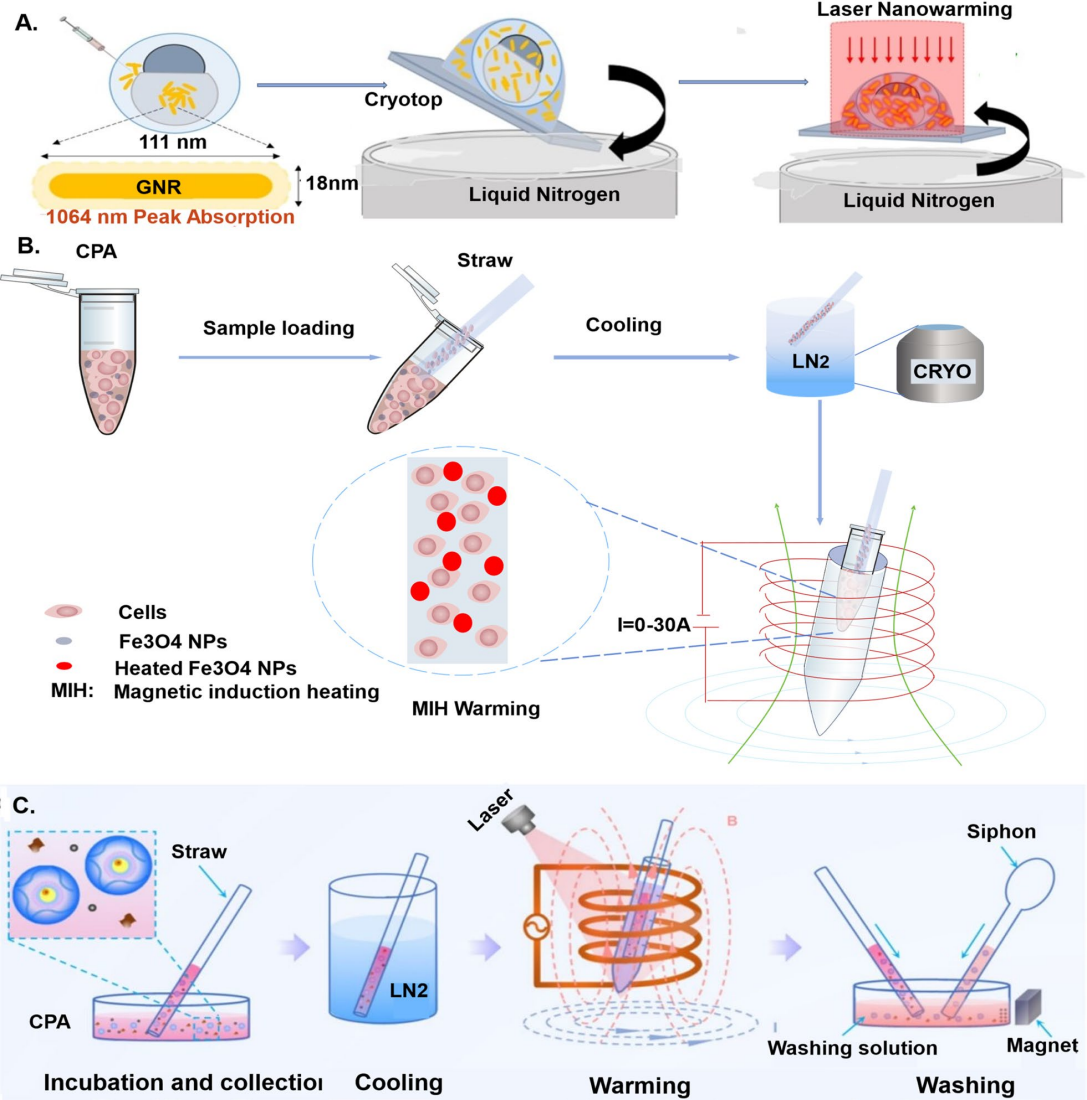
Application and principle of nano-rewarming technology. **A** zebrafish embryo cryopreservation and laser gold nanorods (GNR) rewarming. Laser GNR warming yields rapid and uniform warming inside the embryo to outrun any ice formation [161]. **B** Fe_3_O_4_ nanoparticles for magnetic induction heating (MIH) to enhance rewarming of vitrification-cryopreserved human umbilical cord matrix mesenchymal stem cells (h-UCMMSCs) [164]. **C** Preantral follicles cryopreservation and nanometer warming combining magnetic induction heating (MIH) and laser-induced PAF heating (LIH) [163]

Nanoregulators stand as catalysts at the forefront of fostering enhanced heat generation, marking a transformative chapter in materials science and technology. These minuscule entities, meticulously designed and engineered at the nanoscale, wield remarkable capabilities to harness and amplify heat production with unprecedented precision (Fig. 7).

As early as 2011, Zhang et al. [157] underscored the collaborative potential of nanotechnology and cryopreservation in enhancing the cryopreservation capabilities of small sample volumes. Subsequent studies have demonstrated the distinctive benefits of nanoregulators in both warming and cryoprotection, with magnetic nanoparticles emerging as particularly advantageous in nano-rewarming technology [158, 159]. To illustrate, we present noteworthy examples of nanoregulators in cryopreservation in Table 3. These instances not only showcase the remarkable advancements facilitated by nanotechnology but also prompt a fresh perspective on its application in OTC-T.

**Table 3:** Application of nanoregulators in OTC freezing rewarming

Nanoregulators	Experimental object	Affection	Mechanism	Applied time for rewarming	Refs.
Iron oxide nanoparticles coated with polyethylene glycol silica	Sheep ovary	Prevent apoptosis and oxidative stress and improve follicular development	Rapid and uniform rewarming of magnetic nanoparticles inhibits the formation of ice crystals	Placed in an alternating magnetic field for 1 min	[160]
GNRs	Zebrafish embryo	Significantly improve embryo survival rate	Uniform rewarming of gold nanorods produced by laser pulse irradiation	Laser pulse (300 V power, 1 ms pulse time, I = 1.1 × 109 W m-2 fluence rate), specific action time not mentioned	[161, 162]
GO	Mouse preantral follicles	Significantly improve the survival rate of preantral follicles	The preantral follicles are evenly rewarded by converting light energy into thermal energy under the irradiation of near-infrared laser	Combined MIH and LIH 2.4 s	[163]
Superparamagnetic Fe_3_O_4_ nanoparticles	h-UCMMSC	Significantly improve the cell survival rate and proliferation rate after freezing and rewarming	Fe3O4 nanoparticles generate heat under the action of magnetothermal heating, which uniformly and quickly promote cell rewarming	MIH at a medium frequency (375 kHz) for 10 s	[164]
Magnetic nanoparticles coated with silica Iron oxide nanoparticles (IONPs)	Rat heart	Reduce tissue fracture	Fast and uniform rewarming under alternating magnetic field	Continuous observation for 90 s in a 15 kW, 64 kA m^−1^ and 185 kHz RF coil	[165, 166]

#### Application and principle of nanoregulators as heating agents in the freezing of various cells, tissues, and organs

Magnetic induction heating of magnetic nanoparticles has become a widely employed technique in cell freezing. In 2016, Wang’s team [164] successfully utilized MIH of superparamagnetic Fe_3_O_4_ nanoparticles to significantly enhance the survival rate of h-UCMMSCs after cryo-rewarming. Furthermore, in 2017 and 2020, Khosla et al. [161, 162] conducted experiments incorporating gold nanorods (GNRs) into cryoprotectants for zebrafish embryo cryopreservation and rewarming under nano-laser pulse irradiation. Their findings revealed that nanoparticle-assisted warming markedly improved zebrafish embryo survival compared to the control group.

In 2022, Tian et al. [163] encapsulated mouse pre-sinusoidal follicles in the alginate hydrogel, preserving them in vitrification using a low concentration of cryoprotectant. Employing GO as a heat-conducting material and photosensitizer, they subjected the follicles to 808 nm near-infrared laser irradiation during thawing. This innovative approach resulted in a 33% increase in the survival rate of antral follicles, with the developmental potential and zygote production comparable to the control group.

Nano-warming technology has demonstrated notable advantages in the cryopreservation of mouse pancreatic islets, rat whole liver, and kidneys [167–169]. For instance, in 2022, Gao et al. [165] incorporated silica-coated magnetic nanoparticles of iron oxide (IONPs) into Vitrification Solution 55 (VS55) for cryoprotection in a rat heart model. Subsequent nano-recovery using rapid heating with an alternating magnetic field not only reduced ice crystal formation but also prevented tissue breakage resulting from uneven heat exposure.

In 2023, Joshi et al. [166] delved into the thermo-mechanical stress analysis of different concentrations of silica-coated IONPs for nano-rewarming-assisted recovery in vitrified cryopreservation, focusing on human and rat cardiac models. They point out that variable sIONP concentrations based on cardiac physiology will increase mechanical stress, posing a greater threat to the integrity of organs. However, the nano-warming scheme still helps to reduce the possibility of heart structural damage, and the thermal scheme for heart cryopreservation needs to be tailored to specific conditions such as heart size. The above studies provide more data support for promoting the application of nano-warming technology in OTC-T.

#### Integration of nano-warming technology and OTC

During the thawing process of ovarian tissues, the risk of ice crystal re-formation, mechanical damage, and oxidative stress is heightened if the rewarming is too slow or uneven. This dilemma, where rapid thermal equilibrium occurs at the tissue surface while deeper layers experience sluggish temperature exchange, can adversely impact cellular functions. Addressing this challenge requires more effective rewarming techniques.

Nano-warming technology emerges as a sophisticated approach that leverages magnetic nanoparticles for rapid and uniform rewarming through magnetic induction warming. When subjected to an alternating magnetic field, magnetic nanoparticles become excited, releasing heat to the surrounding tissues as the magnetic field’s direction changes. This dynamic process ensures swift and consistent rewarming, thereby enhancing the freeze–thaw effect.

In a noteworthy study conducted in 2023, Karimi et al. explored a similar avenue by synthesizing iron oxide nanoparticles encapsulated in polyethylene glycolized silica [160]. These nanoparticles were incorporated into the vitrification freezing medium for ovarian tissues in Sheep. Subsequent nano-warming tests revealed that this technique not only mitigates ovarian tissue damage but also improves follicular development, gene expression outcomes, and prevents apoptosis and oxidative stress. The mechanism at play is attributed to the rapid and uniform rewarming facilitated by magnetic nanoparticles, effectively inhibiting ice crystal re-formation. The successful application of nano-warming technology in diverse cellular contexts, tissues, and organs provides compelling experimental support for its implementation in the intricate process of ovarian tissue cryo-warming.

### Assessing the viability of transplanted tissue for survival

In the realm of assessing the viability of transplanted tissue for survival, nanoregulators emerge as pivotal agents, orchestrating a paradigm shift in the precision and efficacy of this evaluation process. These minute entities, meticulously designed at the nanoscale, play a crucial role in monitoring and enhancing the post-transplantation viability of tissues.

One of the remarkable applications lies in the incorporation of nanoregulators for real-time monitoring of cellular activities within the transplanted tissue [170–172]. These nanoscale agents can be tailored to respond to specific biological cues, providing invaluable insights into the dynamic cellular processes essential for tissue survival. The nanoregulators, acting as vigilant sentinels, enable continuous assessments of factors such as oxygen levels, nutrient availability, and overall cellular health, thereby contributing to a comprehensive evaluation of tissue viability.

Furthermore, nanoregulators contribute to the mitigation of post-transplantation challenges, such as inflammation and immune responses. By modulating the local microenvironment and leveraging their unique physicochemical properties, nanoregulators can help create an immunomodulatory milieu, promoting a more favorable atmosphere for transplanted tissue survival. This nuanced control over the immune response enhances the likelihood of long-term viability and integration of the transplanted tissue.

In essence, nanoregulators serve as indispensable tools in the delicate process of assessing the viability of transplanted tissue for survival. Their multifaceted capabilities, ranging from real-time monitoring of cellular dynamics to targeted immunomodulation, underscore their transformative potential in advancing the success and sustainability of tissue transplantation endeavors.

#### Evaluation metrics for tissue viability

Although modern imaging techniques such as ultrasonography and magnetic resonance imaging (MRI) [173] have been employed to assess ovarian viability, the anatomical indicators they monitor, such as follicle number and morphology, do not fully reflect follicular function [174, 175]. Additionally, the analysis of metabolic products often encounters issues with information lag and the presence of false positives and negatives. Currently, the gold standard for evaluating ovarian viability remains histopathological examination. However, this approach undoubtedly causes damage to the ovaries. Therefore, the development of a technique that can assess the viability of transplanted ovaries in an early and non-invasive manner is of paramount importance.

##### In vivo and in vitro viability assessments

The application of nanoregulators in both in vivo and in vitro viability assessments represents a groundbreaking advancement in the realm of tissue evaluation. In in vivo scenarios, nanoregulators play a pivotal role in real-time monitoring, providing dynamic insights into the intricate cellular processes within transplanted tissues. Tailored to respond to specific biological cues, these nanoscale agents enable continuous tracking of crucial parameters, including oxygen levels, nutrient availability, and overall cellular health. The real-time data garnered from in vivo applications of nanoregulators not only enhances the precision of viability assessments but also contributes to a comprehensive understanding of the tissue’s immediate microenvironment and its adaptive responses [176, 177].

In the in vitro context, nanoregulators offer invaluable tools for refining traditional viability metrics. These agents can be employed to simulate and modulate the cellular environment, providing a controlled setting for assessing tissue viability outside the living organism. By influencing factors such as immune response and inflammation, nanoregulators contribute to the creation of more physiologically relevant in vitro models. This enhanced control over the cellular microenvironment ensures a more accurate representation of the tissue’s response, thereby advancing the reliability of in vitro viability assessments.

In essence, the integration of nanoregulators in both in vivo and in vitro viability assessments signifies a transformative leap in the sophistication and precision of tissue evaluation. By bridging the gap between laboratory studies and real-life physiological conditions, nanoregulators pave the way for more accurate, insightful, and clinically relevant evaluations of tissue viability in diverse biomedical applications.

##### Biomarkers for tissue health monitoring

The conventional biomarkers for assessing follicle function mainly include FSH and LH secreted by the pituitary gland, pigment content in follicular fluid, estradiol, follicle size and quantity, AMH, etc. FSH and LH hormones are two key hormones controlling follicle growth and development, assisting in reflecting the functional status of follicles [178]. The level of estradiol is often associated with follicle growth and development. The pigment content in follicular fluid can reflect the maturity and quality of follicles. Follicle size and quantity also reflect ovarian function, and there are differences in follicle size and quantity at different developmental stages. Recent studies have discovered that the assessment of AMH expression on the surface of granulosa cells, as discussed previously in the context of ovarian physiology, offers a prognostic avenue for determining the status of ovarian tissue survival [179, 180]. The targeted examination of AMH expression provides valuable insights into the overall well-being of ovarian tissue.

Furthermore, in the realm of organ transplantation, endeavors have been made to explore angiogenic imaging utilizing nanoparticles endowed with magnetic, fluorescent, and radioactive properties [181]. Building upon this foundation, we propose a novel method employing nanotechnology for the noninvasive monitoring of biomarkers within ovarian tissue. This innovative approach presents a fresh perspective on noninvasive monitoring techniques for assessing the outcomes of ovarian transplantation.

#### Role of nanotechnology in real-time viability monitoring

Nanobubbles (NBs) manifest as spherical nanostructures characterized by surface-active stabilized phospholipid shells enveloping perfluorocarbon gas cores, boasting a diameter of less than 1000 nm. These meticulously designed nanobubbles can undergo modifications, such as the attachment of antibodies or ligands targeting disease-specific molecules. This strategic customization empowers them for applications in early detection and real-time monitoring of diseases at the molecular level, particularly through the innovative modality of ultrasound molecular imaging.

In the realm of ovarian function assessment, AMH [180], a glycoprotein secreted by ovarian granulosa cells, stands out as a significant player. AMH is instrumental in the transformation of pre-sinus follicles to sinus follicles, and it has gained widespread recognition as a specific marker for evaluating ovarian function and independently predicting ovarian reserve function [182]. Against this backdrop, the work of Zhang et al. [137] in 2020 pioneered the creation of AMH-targeted nanobubbles (NBAMH). This involved integrating AMH antibodies onto the surface of nanobubbles. Leveraging ultrasound molecular imaging, the study achieved qualitative and quantitative monitoring of AMH expression, offering a non-invasive means to scrutinize the survival dynamics of transplanted ovaries.

**Fig. 8:**
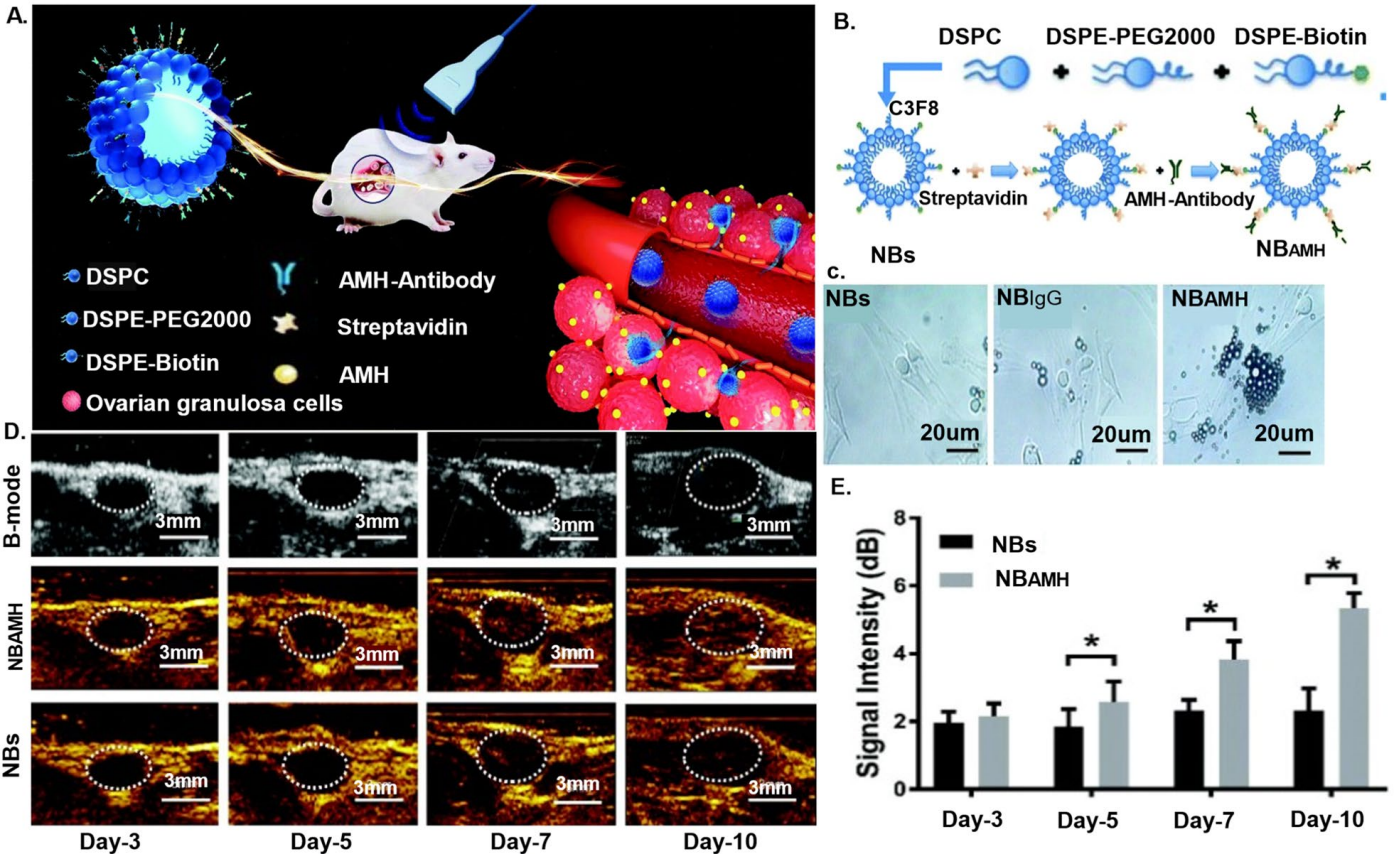
Monitoring the survival rate of ovarian transplantation by AMH targeted nano-bubbles [137]. **A** Schematic of NBAMH and their targeting ability to rat ovarian granulosa cells expressing AMH. **B** Production and purification process of NBAMH. **C** Representative bright-field micrographs of NBs, NB IgG, and NBAMH bound to ovarian granulosa cells. **D** Representative B-mode and ultrasound molecular imaging of ovarian tissue by injection of NBAMH or NBs at different times after transplantation (3, 5, 7, and 10 days). **E** The quantitative analysis of signal intensity from A. Stronger signal intensity was observed in the NBAMH group relative to the NBs group from days 5 to 10 after transplantation. *P < 0.05

The findings revealed a gradual increase in the ultrasound signal of AMH over the transplantation duration, indicative of heightened AMH secretion and progressive tissue survival. Subsequent immunohistochemical staining of ovarian tissue and protein immunoblotting analysis further substantiated these observations. Zhang et al.’s groundbreaking work thus presents an exemplary methodology for the non-invasive monitoring of graft tissue survival (Fig. 8).

Nanosensors, characterized by high sensitivity and rapid response capabilities, have garnered significant attention in recent years, particularly within the biomedical domain. These nanosensors demonstrate the capacity to discern subtle alterations in the intracellular environment, providing a nuanced understanding of cell survival and functionality. A notable instance from 2021 involves the work of Gadly et al. [183], who harnessed carbon nanodots as nanosensors to visualize temperature and pH changes within cancer cells. This application facilitated real-time monitoring of cancer cell survival and guided the administration of anticancer drugs.

In the context of transplanted tissues, the post-transplantation milieu plays a pivotal role in tissue survival and functional recovery. Incorporating nanosensors into Organ Transplantation Therapy holds immense potential for offering real-time insights into critical biochemical and physiological parameters, encompassing pH, ion concentration, and oxygen levels. Leveraging this data enables timely adjustments to treatment strategies, the optimization of post-transplantation care, and the enhancement of overall transplantation success. Looking ahead, nanosensors are poised to emerge as indispensable tools, contributing significantly to the assessment of the overall health and functional recovery process of transplanted tissues.

### Enhancing angiogenesis in transplanted tissue to optimize oxygen deliver

During the early post-transplantation phase, the ovary undergoes ischemic and hypoxic conditions owing to the absence of vascular anastomosis between the transplanted and host tissue. Significant follicle loss occurs during the interval preceding complete hemodialysis of the grafts. Consequently, timely angiogenesis and effective microcirculatory perfusion emerge as pivotal factors for ensuring the survival and functional recovery of transplanted ovaries. In the subsequent discussion, the authors delve into the dual aspects of nanoregulators, focusing on their role in promoting angiogenesis and enhancing oxygen supply to transplanted tissues (Table 4).

**Table 4:** The application of nanoregulators can promote angiogenesis and improve the oxygen supply of transplanted tissue

Nanoregulators	Experimental object	Affection	Mechanism	Refs.
mPEG-PLGA (methoxy poly (ethylene glycol)-poly (lactic-co-glycolic acid)) polymer nanoparticles PLLA poly-l-lactic acid nanofiber and 1.0 wt% graphene oxide	Mouse ovarian tissue	Slow release of NO is related to cyclic guanosine monophosphate dependent or independent pathway	Increase the blood vessel density in the ovary, improve the quantity and quality of healthy follicles after transplantation, maintain the proportion of growing follicles, inhibit follicular activation, and improve the rate of blastocyst formation	[184]
	POI mice and human ovarian tissue	The hydrophilic surface and porous network structure are conducive to cell infiltration, and pe-NO_S_ regulates the trace production of NO in vivo	Promote angiogenesis, improve the fusion of transplanted ovarian cortex and damaged ovarian tissue, increase the number of follicles, and improve ovarian function	[185]
Metal nano-materials (lanthanide elements such as europium)	Transgenic zebrafish embryo	Promote the production of reactive oxygen species, thereby inducing angiogenesis	Promote angiogenesis	[186]
Nickel-titanium NPs	T cell-deficient nude mice	Promote the production of reactive oxygen species, thereby promoting the up-regulation of pro-angiogenic cytokines such as IL-8	Promote angiogenesis	[187]
Oxygen nanobubbles (ONBs)	Dental plaque tissue	Release oxygen, improve cell proliferation under anoxic environment, promotes glucose uptake, inhibit cell apoptosis	Improve the damaged tooth germ	[188]
Micro/nanobubbles (MNBs)	Rat islet cells	High oxygen-carrying capacity can supply oxygen to tissue and improve tissue hypoxia	Adding air-filled MNB is the best way to increase the number and survival rate of islet cells	[189]

#### Nanoregulators as angiogenesis enhancers

In the realm of strategies aimed at promoting angiogenesis in ovarian tissues, traditional approaches encompass the transplantation of ovarian tissues into densely vascularized granulation tissues and freshly decorticated ovarian cortical sites [190]. Additionally, angiogenesis can be induced through the topical or systemic administration of various active substances. These substances comprise vascular endothelial growth factor, follicle-stimulating hormone, erythropoietin, nitric oxide, melatonin, fibroblast growth factor, and stem cells. Certain polymeric materials, such as fibronectin, alginate, collagen, gelatin, and polyethylene glycol (PEG) [191–193], have also demonstrated efficacy in promoting angiogenesis.

However, given the complex nature of ischemic injury, relying solely on a single substance or method often proves to be insufficient. Many active substances encounter challenges such as low bioavailability, inadequate drug concentration at the damaged site, and systemic toxicity when administered. In this context, nanoregulators emerge as promising promoters of angiogenesis. The distinctive properties of nanoregulators enable them to efficiently penetrate damaged tissues, offering enhanced bioavailability and target specificity. Engineered to carry and gradually release specific actives, these materials achieve elevated drug concentrations at the site of damage while mitigating the systemic side effects associated with broad application. Consequently, nanoregulators not only enhance the effectiveness of angiogenic strategies but also unlock novel therapeutic possibilities in the realm of ovarian tissue transplantation.

##### Promotion of blood vessel formation

In the context of Ovarian Tissue Transplantation, NO plays a pivotal role in inducing angiogenesis [194, 195] and exerts a significant influence on follicle and oocyte development [196]. Polymeric nanoregulators, such as PLGA (poly(lactic-co-hydroxyglycolic acid)), PEG, and chitosan, serve as effective carriers for exogenous NO, facilitating the promotion of angiogenesis [197, 198]. These polymeric carriers have demonstrated their efficacy in promoting wound repair, vascularization, and bone regeneration [199, 200].

**Fig. 9:**
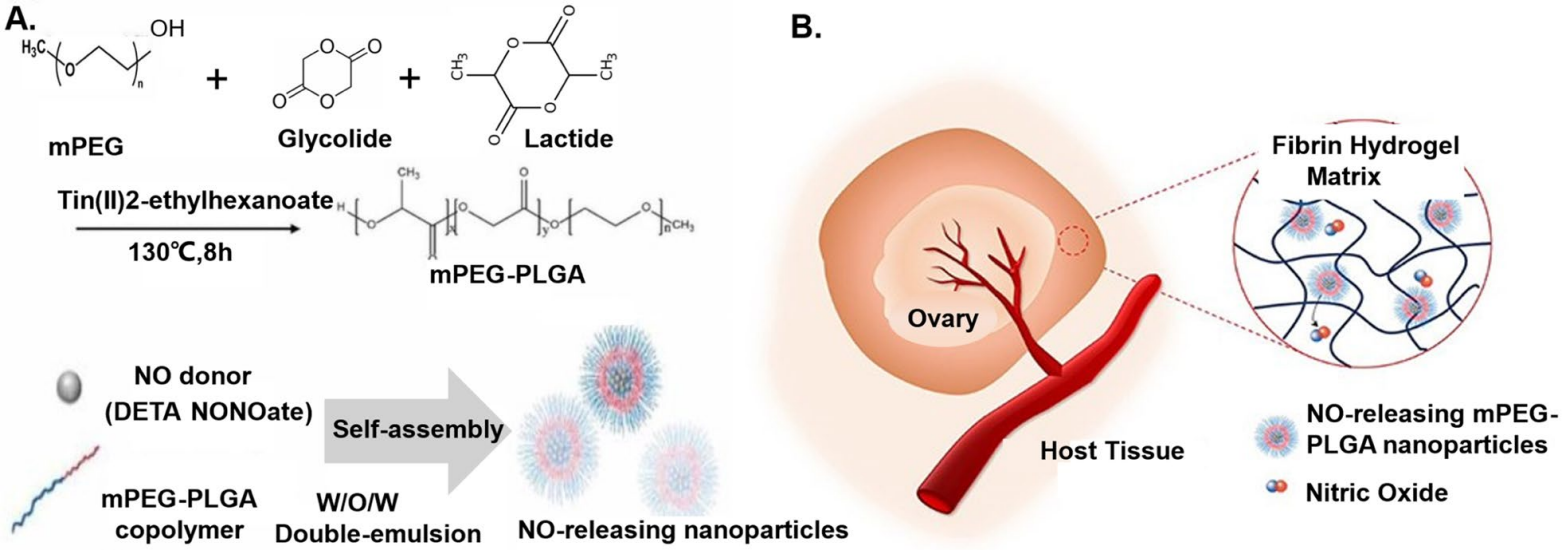
Schematic diagram of the synthesis process and function of NO-releasing PEG-PLGA nanoparticles. **A** Polymerization of mPEG-PLGA from mPEG, lactide, and glycolide. Illustration of NO-NPs by the double-emulsion method containing DETA NONOate as the NO donor; **B** a schematic diagram of the release of NO nanoparticles from the ovary coated with fibrin hydrogel [184]. Nanoparticles release NO into the area around the ovary through the fibrin hydrogel matrix, stimulating blood vessels in the host to produce new blood vessels

A noteworthy example from 2021 involves the work of Yang et al. [184], encapsulated ovaries in a cellular membrane composed of mPEG-PLGA polymer nanoparticles, fibrin hydrogel, and an NO-constructed NO-NP_S_/fibrin hydrogel complex matrix. The controlled release of low concentrations of NO from this complex proved effective in promoting angiogenesis, leading to an improvement in both the total number and quality of follicles in mouse ovarian tissue transplantation. The experimental results demonstrated that the complex matrix provided a conducive growth environment for promoting angiogenesis (Fig. 9).

GO at 10 ng mL^−1^ has been reported to promote cell- and tissue-specific microenvironmental interactions, penetrate cell membranes, and promote cell proliferation, differentiation, and angiogenesis by a mechanism that may be related to the promotion of intracellular reactive oxygen species and reactive nitrogen formation as well as the activation of phosphorylated-Akt and phosphorylated-eNO [201]. Yan et al. [185] constructed a complex fiber scaffold using PLLA poly-l-lactic acid nanofibers with 1.0 wt% graphene oxide, and after co-transplanting this scaffold wrapped with normal ovarian tissue into the in situ ovary of POI mouse model, the fusion of the transplanted ovarian cortex with the damaged ovarian tissues was improved, as well as the ovarian function and follicle number, and they found that the effect of the co-transplantation of GO and PLLA on the survival rate of the ovarian cortex improvement was associated with p-eNO modulation of NO trace production in vivo.

Metallic nanoregulators have been important in the study of promoting angiogenesis due to their specific oxidation state variability and ability to modulate pro-angiogenic signaling pathways. For example, rare earth elements such as europium promote angiogenesis by enhancing intracellular accumulation of reactive oxygen species (e.g., H_2_O_2_) and reactive oxygen species (ROS) around hypoxic regions [186]. On the other hand, main group elements such as nickel enhance the angiogenic process by up-regulating pro-angiogenic cytokines (e.g., IL-8) [187]. Metal nanoparticles such as Europium, Cerium, and Cobalt have been shown to promote angiogenesis by modulating different angiogenic pathways, promoting growth factor production, and reactive oxygen species metabolism [202, 203]. However, the application of these metal nanoparticles in the field of ovarian tissue transplantation has rarely been reported and their potential toxicity needs to be considered when applied to ovarian tissue transplantation.

On the other hand, mesenchymal stem cells (MSCs), as a cell with multidirectional differentiation potential, can differentiate into vascular and related cells. MSC-derived extracellular vesicles (MSC EVs) are nanoscale paracrine mediators secreted by MSCs, which contain a variety of actives that promote angiogenesis and tissue regeneration [204, 205]. In 2022, Sun et al. [206] developed adipose-derived stem cell-engineered nanovesicles, which were found to be superior to conventional MSC EVs in promoting adipose tissue angiogenesis, suggesting their potential as cell-free therapeutic biomaterials in soft tissue regeneration. It is hypothesized that if MSC nanovesicles are applied to ovarian tissue transplantation, the survival rate of transplanted tissues may be significantly enhanced.

##### Improved oxygen supply to transplanted ovarian tissue

In the initial phase following transplantation, the transplanted ovarian tissue often experiences ischemia and hypoxia due to the absence of vascular anastomosis between the graft and host tissues [207]. This condition, exacerbated by the lack of adequate oxygen supply, can lead to substantial follicle loss during the complete hemodialysis of the grafts. Ensuring a sufficient and timely oxygen supply to transplanted ovarian tissues is critical for their survival and functional recovery.

Nanoregulators offer a promising avenue to address this challenge and enhance oxygen supply to transplanted ovarian tissue [188, 189]. Through innovative applications of nanotechnology, these regulators can be designed to facilitate improved oxygen delivery and distribution within the tissue microenvironment. Their unique properties, such as the ability to penetrate tissues effectively, allow for targeted interventions to enhance oxygenation [208].

By leveraging nanoregulators, it becomes possible to tailor oxygen-carrying capacities, optimize release kinetics, and enhance the overall oxygen availability in the microenvironment of transplanted ovarian tissues [209]. This approach holds significant potential for mitigating the ischemic conditions, thereby promoting tissue survival, maintaining functionality, and ultimately improving the success rates of ovarian tissue transplantation [210]. The integration of nanoregulators into strategies aimed at enhancing oxygen supply represents an innovative and promising direction in the field of ovarian tissue transplantation.

### Mitigating ischemia–reperfusion damage and oxidative stress

In the context of ovarian tissue transplantation, the occurrence of ischemia–reperfusion following revascularization initiates an escalation in ROS, inducing oxidative stress and exacerbating tissue injury—an additional significant factor contributing to follicle loss [211]. While numerous studies have explored the application of nanotechnology to mitigate ischemia–reperfusion injuries in organ allografts such as the heart, liver, and kidney [212–214], there is a scarcity of reports on the utilization of nanoregulators specifically tailored for OTC-T. In this discussion, we highlight currently documented nanoregulators that exhibit potential relevance to OTC-T, offering insights into their potential applications across various domains. This serves to stimulate comprehensive consideration and exploration of their application in the context of ovarian tissue transplantation (Table 5).

**Table 5:** Application of nanoregulators to reduce tissue ischemia reperfusion

Nanoregulators	Experimental object	Affection	Mechanism	Refs.
Micro/nano motor-MG micromotor	Arthritic rats	Improve joint injury and inhibit the severity of overall arthritis	Hydrogen produced by magnesium contact with water pushes the motor forward; hydrogen removes inflammatory substances and reactive oxygen species from tissues and reduces oxidative stress damage to tissues	[215]
	Middle cerebral artery occlusion in rats	The area of cerebral infarction was significantly reduced and the ability of spatial learning and memory was improved in rats	Mentioned above	[216]
Nano-enzyme: cysteine copper nano enzyme coated with albumin	Sperm	Sperm motility increased and apoptosis decreased	Scavenging ROS and reducing oxidative stress produced by reactive oxygen species	[217]
Ultraminiature Cu5.4O-based nanoparticles	Mouse	Significantly improve the therapeutic effects of various ROS-mediated injuries such as acute renal injury, acute liver injury, and wound healing	At the same time, it has the characteristics of catalase, superoxide dismutase, and glutathione peroxidase mimics enzymes and efficiently scavenges ROS	[218]
New gold and platinum nanoparticles	Renal ischemia–reperfusion injury model in mice	Attenuate ischemia–reperfusion injury in mice	Through the release of oxygen, it can improve cell proliferation, apoptosis, and glucose uptake under hypoxia	[219]

#### Nanoregulator strategies for ischemia–reperfusion injury

Strategies involving nanoregulators have emerged as promising avenues to address the challenges posed by ischemia–reperfusion injury, particularly in the realm of OTC-T. Ischemia–reperfusion-induced ROS elevation contributes significantly to oxidative stress, exacerbating tissue damage and follicle loss [211]. While existing research extensively explores nanotechnology’s role in mitigating ischemia–reperfusion injuries in various organ allografts, the application of nanoregulators in the context of OTC-T is still a developing area.

Several nanoregulators have demonstrated efficacy in reducing ischemia–reperfusion injuries, offering potential avenues for improving OTC-T outcomes. These nanoregulators, with their unique properties, hold promise in minimizing oxidative stress and enhancing tissue resilience during the critical phases of ischemia and reperfusion. Comprehensive exploration and understanding of these nanoregulator strategies could pave the way for innovative approaches to tackle ischemia–reperfusion injury in ovarian tissue transplantation, potentially improving the success rates and overall efficacy of the procedure.

As a burgeoning drug delivery system, micro/nano motors have garnered substantial research attention in recent years owing to their remarkable penetration ability, ample power supply, and high-efficiency characteristics [220, 221]. The field has seen accelerated progress, especially following the demonstrated potential of hydrogen in treating diseases associated with heightened ROS [222, 223]. However, the therapeutic efficacy of hydrogen is hindered by its low retention and solubility in the body.

**Fig. 10:**
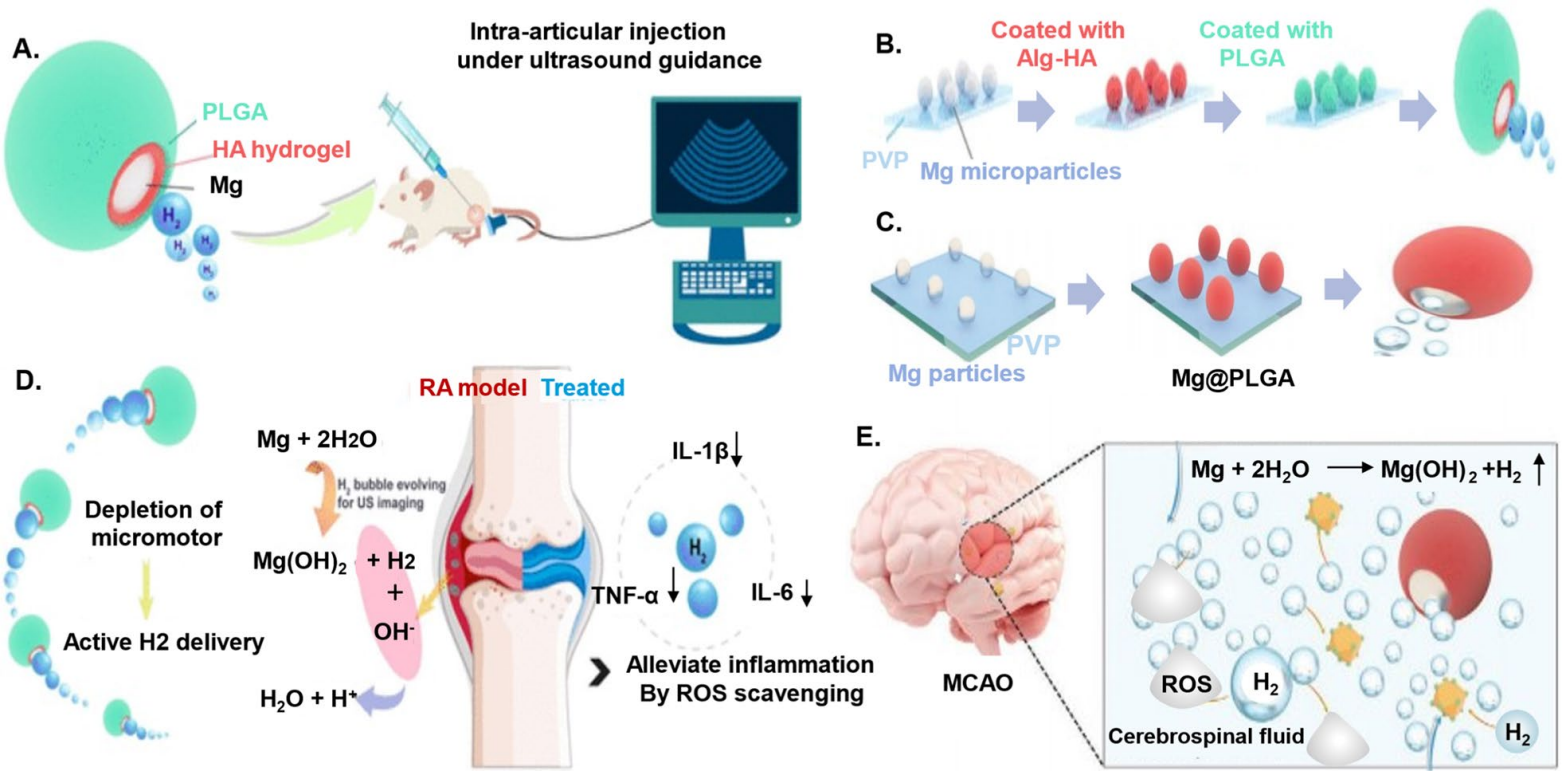
Self-produced hydrogen gas-powered magnesium micro-motor for the treatment of rheumatoid arthritis and acute ischemic stroke [215, 216]. **A** Schematic diagram of Mg-HA motor driven by self-produced hydrogen actively producing hydrogen under the guidance of ultrasound for RA treatment. **B** Schematic illustration of the fabrication of Mg–HA motors. **C** Schematic illustration of HPMs fabrication. **D** Mechanism of the H_2_-mediated RA curative effect by Mg–HA motors. Active H_2_ alleviates RA progression by ROS scavenging and further attenuates inflammation along with inflammatory cytokines. **E** Schematic illustration of HPMs as in situ H_2_ generator for ischemic stroke remedy by active H_2_ delivery to scavenge ROS and alleviate oxidative stress

Addressing this challenge, Xu et al. [215] pioneered the integration of micro- and nano-motors with hydrogen therapy in 2021 by developing MG micromotors capable of autonomously producing and continuously releasing hydrogen (Fig. 10). The hydrogen, generated through the reaction between magnesium and water, not only propelled the motor forward but also efficiently scavenged inflammatory substances and reactive oxygen species, thereby reducing oxidative stress damage to tissues. Furthermore, the ultrasound monitoring of generated hydrogen bubbles enabled precise in vivo localization. In the same year, Wang et al.’s team [216] successfully treated acute ischemic stroke in rats using hydrogen generated by magnesium-based micromotors. These studies underscore the significant potential of magnesium-based micro-nanomotors for the controlled release and diffusion of hydrogen, offering promising applications in mitigating the consequences of oxidative stress.

Meanwhile, nanoenzymes, as a fusion of nanomaterial properties and mimetic enzyme catalytic function, have also shown a wide range of potential applications in several fields [224, 225]. Their effective scavenging ability of reactive oxygen species has been particularly emphasized concerning their use as mimetic antioxidants. For instance, Dashtestani’s team [217] achieved significant results in reducing oxidative stress during sperm cryopreservation by employing albumin-coated copper cysteine nanoenzymes in 2018. In 2020, Liu et al. [218] developed ultra-small copper-based nanoparticles (Cu5.4O USNPs), exhibiting excellent ROS scavenging ability and biocompatibility in the treatment of acute kidney injury, acute liver injury, and wound healing. In 2022, Feng et al. [219] found that gold-platinum nanoparticles, acting as nano-enzymes, served as broad-spectrum antioxidants and effectively attenuated renal ischemia–reperfusion injury. These advances underscore the significance of nanoenzymes in biomedical applications, particularly in antioxidant therapy.

Additionally, certain pro-angiogenic substances face limitations due to factors like hydrophobicity; however, ROS-responsive nanomedicines have shown promising results in treating ischemia–reperfusion injury across various tissues and organs [226]. While studies employing nanomotors, nanoenzymes, and ROS-responsive drug release technologies in the field of ovarian tissue transplantation are relatively scarce, their application presents new perspectives to enhance the success of OTC-T.

### Removal of tumor cells from ovarian tissue

While ovarian tissue transplantation has demonstrated its efficacy in restoring hormonal cycles and fertility [227], particularly in cases of hematologic and ovarian malignancies, there remains a potential risk of malignant contamination within the transplanted tissue [228]. This introduces the possibility of tumor recurrence upon reintegration into the recipient’s body. Human and mouse models of hematological malignancy transplantation have provided insights into this concern [229–231]. Addressing tumor reinfection in these patients can involve alternative approaches such as the use of artificial ovaries, stent grafting of individual follicles, or in vitro maturation of pre-sinus follicles [232]. Moreover, emerging research indicates that leveraging nanotechnology for the targeted removal of tumor cells from tissues presents a potentially simpler, safer, and more accessible strategy to reduce the risk of tumor reinfection.

#### Tumor cell clearance strategies

**Fig. 11:**
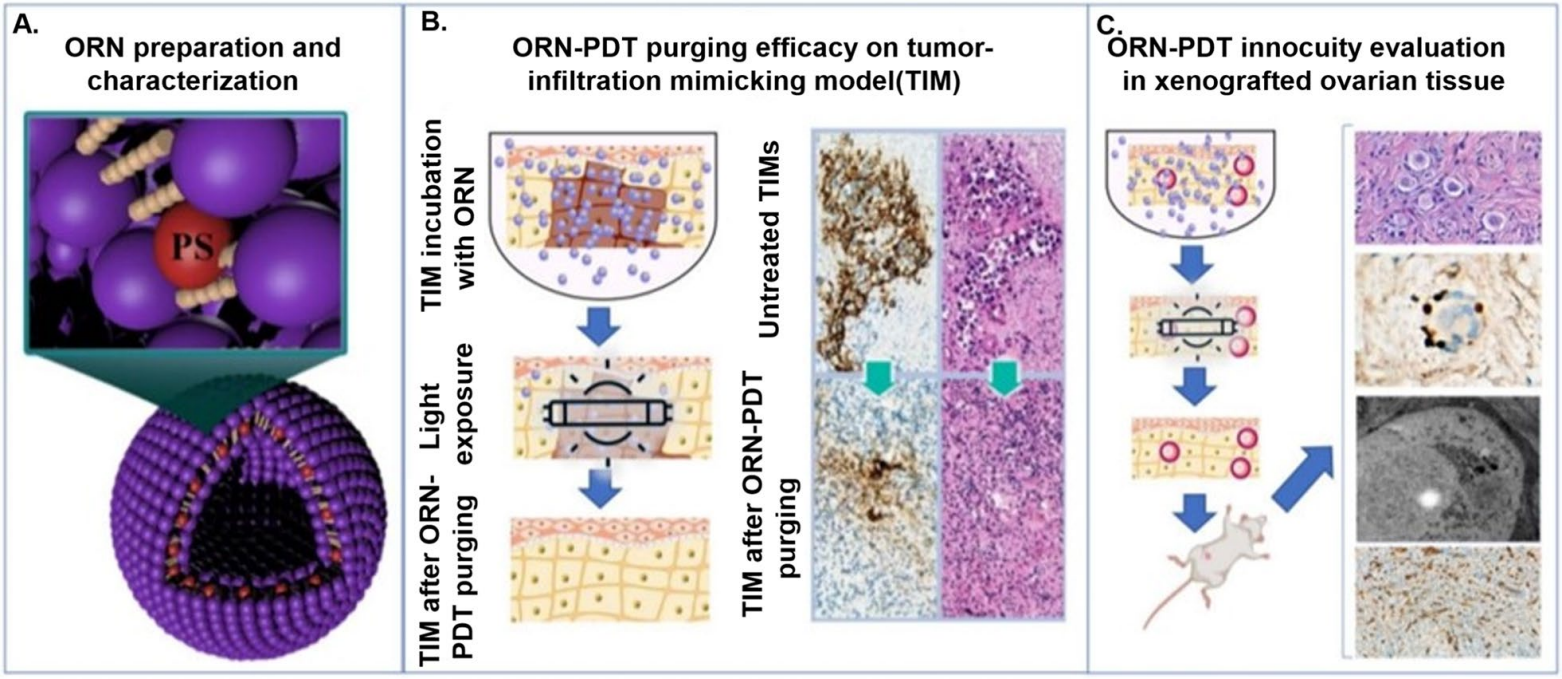
Nano-vesicles loaded with OR141 successfully eliminated chronic myeloid leukemia cells from sheep ovarian fragments by PDT in vitro [234]. **A** Preparation and characterization of vesicles containing OR-141 (ORN). **B** Evaluation of the efficacy of ORN–PDT using tumor models in ovarian tissue fragments; and **C** evaluation of PDT specificity using ovarian biopsies from young patients

Moghassemi’s team [233–235] conducted a significant endeavor, as illustrated in Fig. 11, showcasing a novel approach to address this concern. Photodynamic therapy (PDT) stands out as a therapeutic method utilizing photosensitizers in conjunction with light energy to obliterate cells [236, 237]. In this process, the photosensitizer accumulates in target cells, and upon activation by light at specific wavelengths, induces substantial cytotoxicity in the presence of oxygen, thereby causing cellular damage.

The nan-porphyrin drug OR141 proves particularly effective in the hypoxic environments of tumors. Functioning as a photosensitizer, OR141 induces immunogenic cell death by oxidizing endoplasmic reticulum-associated proteins upon photodynamic activation [238, 239]. Nanovesicle (NVS), a vesicle structure synthesized with nanoregulators like liposomes or quantum dots, exhibits non-immunogenic and biocompatible properties. When affixed with targeted drugs, NVS enhances the bioavailability and efficiency of OR141, overcoming limitations associated with limited tumor tissue aggregation and tissue penetration of OR141.

In light of these considerations, Saeid Moghassemi et al. meticulously designed, optimized, and synthesized nano-liposomal vesicles loaded with OR141. Through PDT, this innovative approach successfully eradicated chronic myeloid leukemia cells from sheep ovarian fragments in vitro (Fig. 11). Importantly, this therapy demonstrated a lack of damage to ovarian mesenchymal cells. In 2023, Moghassemi [234] assessed the purification efficiency of this method and conducted mouse xenografts. The findings indicated that this approach efficiently removes tumor cells without compromising follicular survival, development, and ovarian quality.

In recent years, a multitude of strategies have been employed to eradicate tumor cells within ovarian tissue [97, 240, 241], the light-dependent nature of PDT suggests its potential safety superiority over other methods. Even if the photosensitizing agent remains in the body after transplantation, PDT is not toxic to the organism.

## Challenges and solutions in clinical application

Despite the vast potential of nanomodulators in the field of ovarian cryopreservation and transplantation, there are still some challenges that hinder their seamless clinical application. A primary concern is that certain nanoregulators may induce toxic damage to tissue organs, particularly regarding reproductive toxicity. Therefore, achieving a delicate balance between the seamless integration of nanoregulators with OTC-T and avoiding reproductive toxicity remains a key challenge.

### Difficulties in applying nanoregulators to clinical settings

Primarily, OTC-T stands as an emerging field, with insufficient research on the widespread use of nanoregulators in OTC-T. The majority of experimental evidence originates from non-human studies, necessitating further research and practical applications to establish the feasibility and safety of their implementation in the human body. Nanoregulators encounter various challenges, encompassing issues like toxicity damage, biological feasibility, intricate distribution and metabolic pathways within the body, lack of standardization and regulation, and even ethical considerations, such as those related to gene editing.

Nanomaterials within biological systems may undergo various transformations and interactions, including absorption, phagocytosis, or adsorption onto cell surfaces, followed by internalization into cells or entry into other organs via the lymphatic and bloodstream circulation [242]. Moreover, different tissues and cells may utilize distinct metabolic pathways, such as lysosomal degradation, hepatic transformation, or renal excretion. For instance, TiO_2_ nanoparticles can be absorbed through the skin, and undego metabolism and excretion by the liver and kidneys, while quantum dots can disseminate via the lymphatic system and accumulate in lymphoid organs.

Current research primarily focuses on optimizing the size, shape, surface modifications, and chemical composition of nanomaterials to reduce uncertainties in their metabolism and distribution [243]. Simultaneously, a comprehensive understanding of nanomaterial behavior within biological systems is pursued through high-resolution imaging techniques, bioinformatics analysis, and methods such as Nano-QSAR computational modeling to predict nanoparticle cytotoxicity [244, 245]. These approaches aim to assess the safety and efficacy of nanomaterials in clinical applications.

**Fig. 12:**
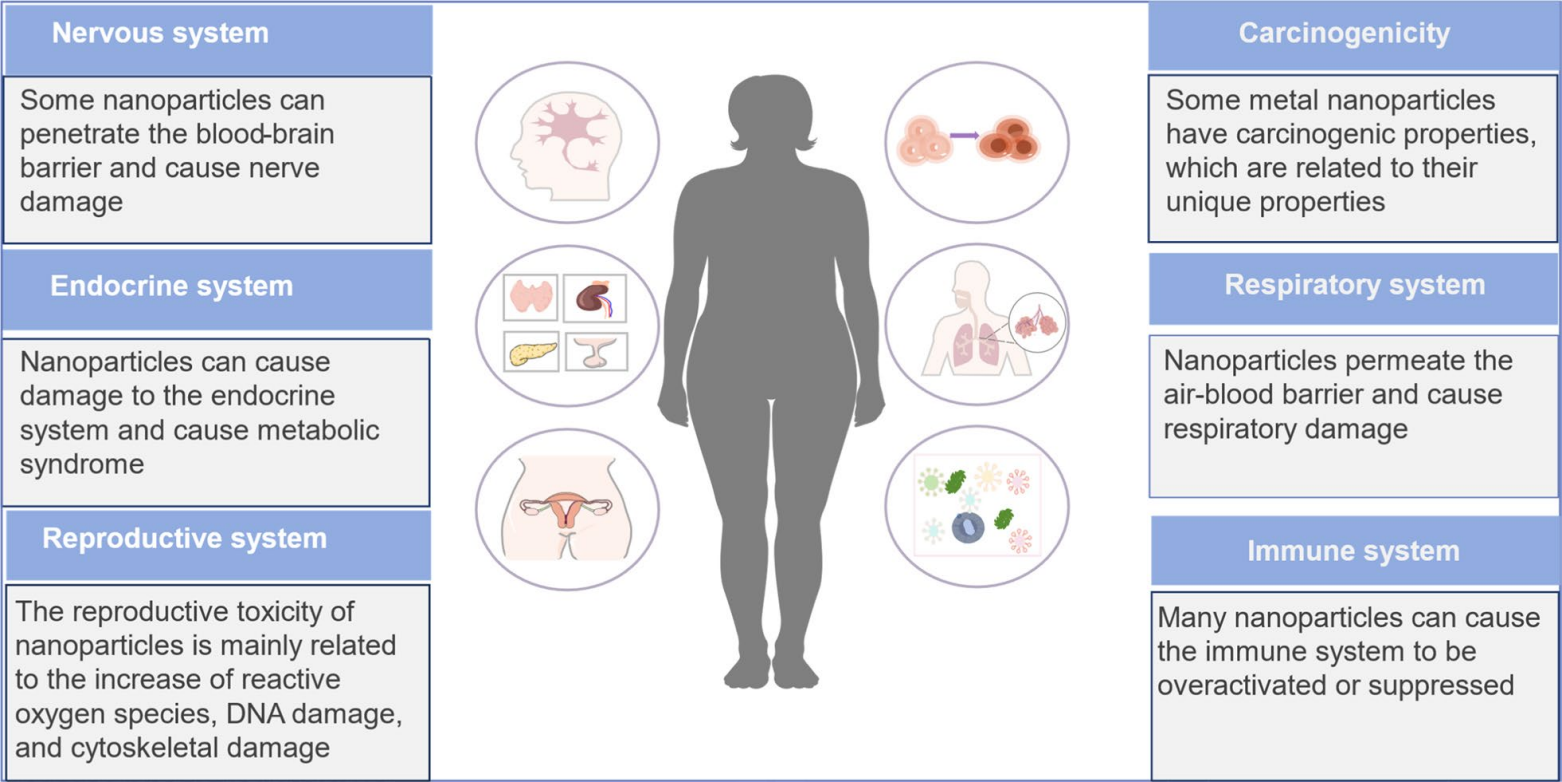
Damage to various systems by nanoparticles

Secondarily, while the clinical advantages of nanotechnology may outweigh many associated risks, nanoregulators are not exempt from causing toxicity damage to multiple systems within the human body, including the respiratory, nervous, endocrine, and reproductive systems [246] (Fig. 12). Risks linked to nanoregulator toxicity stem from their highly reactive nature, prolonged circulation time, accumulation of off-target nanoregulators, and inadvertent exposure [247–249]. Some nanoregulators are also inherently carcinogenic, with reproductive toxicity, in particular, standing out as a significant area of concern.

In 2024, Waseem Ali provided an overview of the adverse effects of micro/nanoplastics on the reproductive systems of both male and female mammals as well as aquatic organisms [250]. They concluded that micro/nanomaterials can impact the hypothalamic–pituitary axis, leading to oxidative stress, reproductive toxicity, neurotoxicity, cytotoxicity, developmental abnormalities, poor sperm quality, reduced ovarian ovulation, and immunotoxicity. Furthermore, they affect numerous signaling pathways associated with male and female reproductive and developmental toxicity, such as the MAPK signaling pathway and oxidative stress, Fibrotic signaling pathways and NLRP3, Apoptosis signaling pathway, among others.

**Fig. 13:**
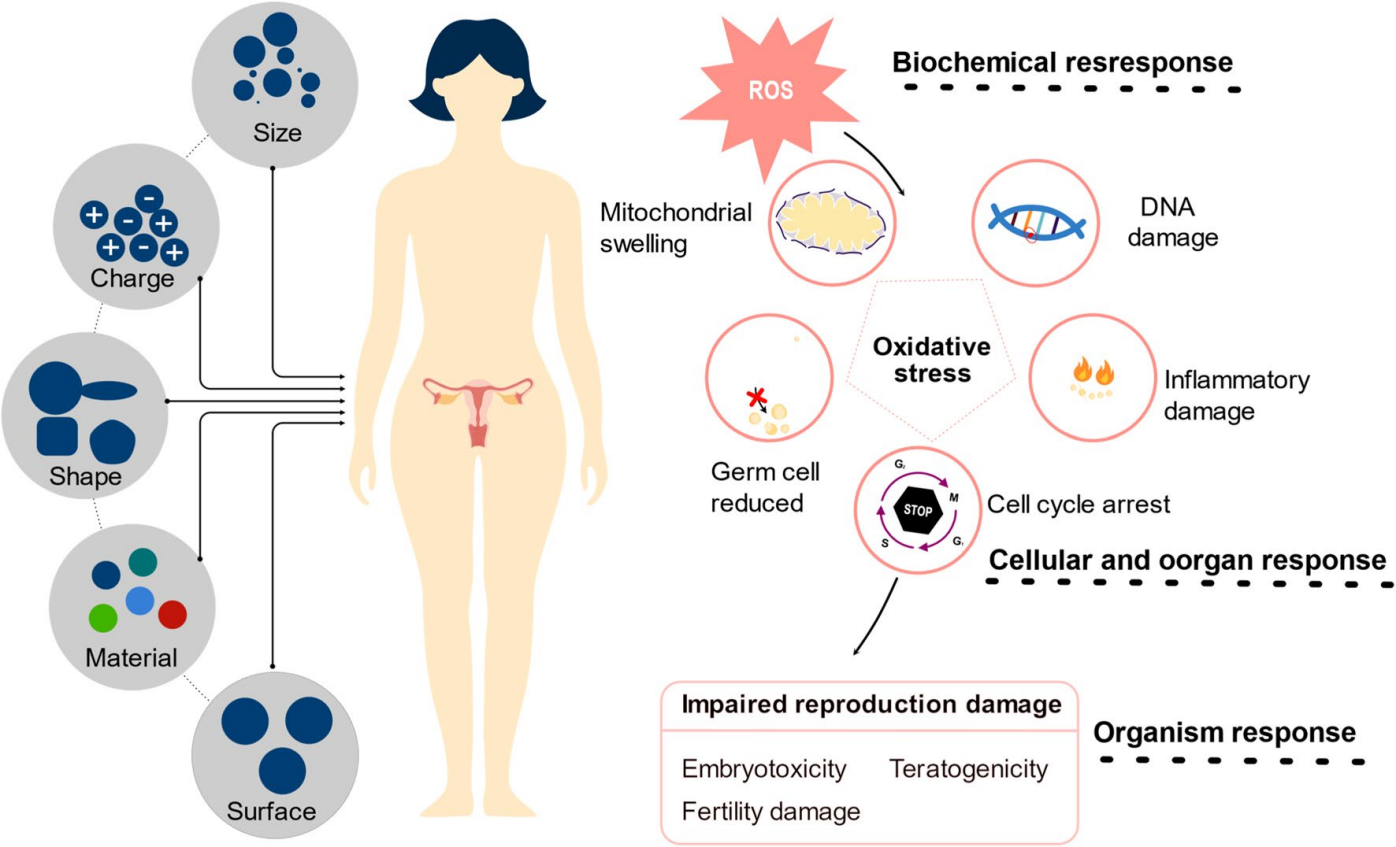
The main mechanism of toxic damage of nanoparticles to the female reproductive system. The toxicity of nanoparticles is mainly related to the size, charge, and shape of nanoparticles, which produce a large amount of reactive oxygen species to cause oxidative stress, resulting in mitochondria, DNA damage, cell cycle arrest, and so on, resulting in impaired reproductive damage

The deleterious impacts of nanoregulators, particularly metal nanoparticles, on the reproductive system are considerable. Research indicates that the toxic effects of nanoparticles on the female reproductive system are predominantly linked to their physical properties, such as size, charge, and shape. These nanoparticles can generate an abundance of reactive oxygen species, instigating oxidative stress. This, in turn, results in a cascade of biological effects including mitochondrial damage, DNA damage, and cell cycle blockage, ultimately disrupting the normal functioning of the reproductive system [30] (Fig. 13).

For example, in 2015, Zhao and their team conducted an exhaustive analysis and summary of the top 20 pathways identified in KEGG analysis, coupled with an assessment of the overall gene and protein expression affected by ZnO-NP treatment at 5 μg/mL [251]. They observed that while the specific pathways responsible for the effects remain to be precisely determined, ZnO-NPs exert a targeted modulation of gene and protein expression in ovarian granulosa cells, inhibiting their proliferation and consequently inflicting damage on the female reproductive system. In a separate study in 2021, Saber et al. demonstrated the concentration and time-dependent cytotoxicity of ZnO-NPs on mouse ovarian germ cells. Low concentrations failed to induce significant cytotoxicity, but heightened concentrations, reaching up to 30 µg/mL, or prolonged exposure for 7 days, resulted in pronounced cytotoxicity, accompanied by a concentration-dependent increase in intracellular ROS levels. This cytotoxicity is primarily mediated through the induction of oxidative stress, a key mechanism underlying the toxicity of metal oxide NPs [112]. Subsequently, Mawed SA’s team, in 2022, uncovered that ZnO-NPs impede zebrafish oocyte development by instigating autophagy, apoptosis, and oxidative stress processes [113].

Additionally, titanium dioxide nanoparticles are renowned for their adverse effects on embryonic and follicular development [252]. In a study from 2019, Zhou et al. administered various doses of TiO_2_ NPs intragastrically to female mice over 60 consecutive days. They observed inhibition of normal ovarian follicle development, attributable to dysfunction in TGF-β, PI3K/AKT/mTOR, and AKT/p70S6K-rpS6/TSC/mTOR pathways [253]. Similarly, in 2023, Ji et al. exposed female mice to varying concentrations of TiO_2_ NPs via gavage for 60 days. They observed elevated levels of FSH and LH hormones, increased expression of molecules associated with follicular development, and a reduction in ovarian weight, all through the TGF-β signaling pathway [254].

Moreover, non-metallic nanomaterials, such as polystyrene nanoparticles (Nano-PS), have shown reproductive toxicity. In a study from 2023, Huang et al. investigated the effects of Nano-PS by administering different concentrations to mice for 8 weeks and conducting in vitro experiments on COV434 cells. The results revealed a decrease in ovarian reserve, elevated oxidative stress and apoptosis, and disruptions in the estrous cycle, implantation rate, and litter size. Furthermore, Nano-PS induced cytotoxic effects on COV434 cells, validating its mechanism of reproductive toxicity through oxidative stress induction [255].

The reproductive toxicity of nanoparticles underscores the imperative of their biosafety. Future research will focus on unraveling the mechanisms of nanoparticle toxicity, particularly their impacts on the reproductive system, and strive towards the development of safer nanomaterials. Additionally, a meticulous assessment of the potential effects of nanomaterials on various systems is essential to ensure their sustainable application in medicine and biology. Moreover, increased attention to the distribution and metabolic kinetics of nanoparticles in organisms will facilitate a more comprehensive evaluation of their potential toxicity.

### Proposed solutions for effective integration

There are many other cases like this, so the application of nanoregulators in OTC-T needs to be considered carefully, both to utilize its unique advantages to solve the challenges in OTC-T and also to conduct further research to obtain more data to support the feasibility and safety of its application in OTC-T. The toxicity of nanoparticles mainly depends on their physicochemical properties [256, 257] such as particle size, shape, surface chemistry (charge, ligands) [258–260]. Therefore, altering these parameters to formulate nanoregulators with unique properties without toxicity is a worthy area of research. In recent years, some studies have proposed solutions such as the development of microbial-derived nanoregulators, optimization of nanoparticle synthesis techniques and material selection, naturally derived cell membrane encapsulation strategies, shape modification of nanoparticles [261], surface modification of protective coatings, and introduction of functional groups to alter hydrophobicity and compatibility [262, 263]. For instance, magnetic nanoparticles exhibit immense promise in diverse interdisciplinary domains such as drug delivery and magnetic resonance imaging, notwithstanding their notable cytotoxicity. Therefore, exploring diverse methodologies to enhance their biocompatibility becomes imperative.

In 2023, Christian Chapa González and colleagues discerned that the cellular compatibility of cobalt ferrite nanoparticles could be augmented through chemical substitution and surface modification utilizing aminosilanes [264]. Similarly, in 2024, Ana Todorović’s team succeeded in mitigating the reproductive toxicity of TiO_2_ nanoparticles in rats through surface modification employing salicylic acid and 5-aminosalicylic acid. These endeavors exemplify the ongoing quest to refine nanoregulators, underscoring the continuous pursuit of safer and more efficacious applications in the realm of nanotechnology [265].

In addition to this, exploring more green and nontoxic Nanoregulators is also an effective alternative [266]. Next, we outline an environmentally friendly and non-hazardous application of nanotechnology: microbial-derived nanoregulators. The research and development of this reactor is intended to guide and expand the exploration of avenues to reduce the potential toxicity of nanoregulators. Through this approach, we expect to provide new ideas and solutions for the safety and environmental friendliness of future nanotechnology.

#### Proposing microbial-derived nanoregulators

Nanoparticles are typically synthesized using one of three primary methods: chemical, physical, or microbial. The physical and chemical synthesis techniques often necessitate toxic chemicals, leading to the potential for “nanotoxicity” due to residue or adsorption of these substances on the nanoparticles’ surface. This toxicity can harm cells and tissues.

Physicochemical synthesis techniques are commonly employed in nanoparticle preparation, often requiring the assistance of organic or inorganic compounds as stabilizers. Among these, certain stabilizers exhibit exceptional biocompatibility. For example, in 2024, the latest research conducted by Yuan and colleagues unveiled the utilization of a covalent organic framework with remarkable biocompatibility as a stabilizer for the synthesis of ultra-small silver nanoparticles [267]. Furthermore, numerous other stabilizers have demonstrated favorable biocompatibility, including polyethylene glycol (PEG), gelatin, and polyvinyl alcohol.

Nevertheless, the majority of stabilizers, including certain metal ions [268] and dimethyl sulfoxide [269], may present some degree of cytotoxicity. The presence of these substances as residues or adsorbates on the surface of nanoparticles may give rise to “nanotoxicity,” thereby causing damage to cells and tissues, significantly constraining the biological applications of nanoparticles.

Moreover, certain nanoparticles may be intrinsically toxic. Of particular concern is the toxicity to germ cells, which exhibit low tolerance to such toxicity. Addressing the mitigation of nanomaterial toxicity has thus become a pivotal area of research in the field of nanotechnology. The pursuit of safer, non-toxic methods for synthesizing nanoregulators, along with strategies to minimize their toxicity, has gained significant research interest. An emerging approach is the biosynthesis method, wherein microorganisms such as bacteria, algae, fungi, and yeast cells act as reducing or stabilizing agents [270–273]. This method facilitates the production of biocompatible nanoparticles, as evidenced in Kim et al.’s 2018 study [274]. Bio-nanoparticles produced via this technique show promise in biomedical applications, including targeted drug delivery, tissue engineering, and biosensors, owing to their non-toxic nature and superior biocompatibility [275]. In this context, we explore the applications of bio-nanoparticles in various fields, particularly focusing on their potential for nanotechnology applications. This discussion aims to present novel ideas and solutions for addressing the toxicity challenges associated with nanoregulators in these areas.

##### Unique mechanisms of microbial nanoregulators

Microbial nanoregulators harness various microorganisms, including bacteria, fungi, and yeast, for nanoparticle synthesis [276]. These organisms facilitate the nucleation and aggregation of nanoparticles through biochemical mechanisms, such as oxidation/reduction reactions, electron transfer, and ionic transformations. This process results in the formation of nanoparticles characterized by excellent biocompatibility [277]. These nanoregulators are capable of synthesizing not only single metals and metal oxides but also complex structures like bimetallic or polymetallic nanoparticles. The synthesis predominantly involves metal ion trapping, enzymatic reduction, and nanoparticle capping [278]. During biosynthesis, the electronegative cell surface of microorganisms enables strong adsorption and uptake of metal cations. Metal ions are initially captured on or within microbial cells, and then reduced to nanoparticles in the presence of specific enzymes. These enzymes serve dual roles: they provide nucleation sites and electrons for metal reduction, and they influence nanoparticle formation through the generation of organic polymers. These polymers modulate the stability of metal ions, thus impacting the nucleation process.

##### The potential of microbial nanoregulators for OTC-T applications

Although the application of microbial nanoregulators in OTC-T has rarely been reported, an overview of its application in related fields can enlighten its potential application in OTC-T. The biosynthesis method shows significant advantages in the synthesis of nanoregulators, especially in terms of reducing the dependence on harmful chemicals, thus becoming a sustainable and green alternative [279, 280]. Nanoparticles synthesized by this method are usually attached with a lipid molecular layer, which not only effectively mitigates the potential toxicity of the nanoparticles, but also significantly improves their solubility, biocompatibility and stability. Therefore, the application of bio-nanoparticles in OTC-T can reduce concerns about the toxicity of nanoregulators and promote their use in OTC-T. The application of bio-nanoparticles in bio-imaging has also received much attention. It has been found that certain bio-nanoparticles exhibit excellent fluorescence properties, which, combined with their good biocompatibility, enable them to easily penetrate cell membranes and enter the cell interior. As a result, these particles are useful in bioimaging [281, 282], and when applied to OTC-T, they can image the cellular interior more efficiently than other nanoparticles, thus providing a more accurate guide for monitoring the survival of transplanted tissues. In addition to this, bio-nanoparticles have significant antioxidant activity and can efficiently scavenge excess reactive oxygen species from tissues [283–285]. The application of these particles in OTT is expected to significantly improve the graft success rate. Bio-nanoparticles are also prominent in tissue engineering, e.g., bio-nanoparticles can form nanofibrillar scaffolds, which have been shown to be effective in repairing and improving damaged tissues and organs. Such as the regeneration of skin and blood vessels [286]. It is expected to improve fertility preservation outcomes if applied in techniques such as artificial ovary construction and ovarian tissue in vitro culture systems.

## Future and outlooks

Nanoregulators stand at the forefront of groundbreaking advancements in OTC-T, playing a pivotal role in reshaping the landscape of reproductive medicine. The significance of nanoregulators in this context cannot be overstated, as they offer a unique and innovative approach to address challenges associated with cryopreservation and transplantation. These challenges, ranging from tissue damage to functional impairments, underscore the critical need for transformative solutions to safeguard reproductive capacity and combat disorders within the female reproductive system.

Navigating the intricate terrain of cryopreservation and transplantation challenges, nanoregulators emerge as promising solutions. This section delves into the multifaceted functions and mechanisms of the proposed nanoregulator, meticulously designed to meet the specific requirements of ovarian tissue preservation and transplantation. By systematically addressing the intricacies of the physiological structure and microenvironment of the ovary, this review lays the groundwork for understanding how nanoregulators can effectively mitigate tissue damage and enhance overall transplant success. However, owing to safety concerns, the application of nanomodulators in clinical practice remains restricted.

To effectively harness nanomodulators derived from clinical microorganisms, innovative strategies have been proposed. This review explores the transformative potential of nanoregulators sourced from microbial origins. By harnessing the power of microbial-derived nanoregulators, we anticipate breakthroughs in therapeutic interventions. The evolution of these innovative methods is poised to deepen the integration of nanoregulators, marking a paradigm shift in the dynamic restoration of ovarian tissue. This visionary approach heralds a future where microbial-derived nanoregulators play a central role in advancing the field.

In conclusion, this comprehensive review serves as a cornerstone in understanding and advancing the role of nanoregulators in ovarian tissue cryopreservation and transplantation. By elucidating their critical importance, addressing current challenges, and envisioning future applications, the review sets the stage for a transformative era in reproductive medicine. The proposed nanoregulator design, coupled with the exploration of microbial sources, signifies a promising trajectory towards enhanced success rates and expanded prospects in ovarian tissue preservation and transplantation. This synthesis of knowledge not only contributes to the scientific discourse but also holds profound implications for the future of female reproductive health and well-being.

## Data Availability

Not applicable.
